# Performance assessment of variant calling pipelines using human whole exome sequencing and simulated data

**DOI:** 10.1186/s12859-019-2928-9

**Published:** 2019-06-17

**Authors:** Manojkumar Kumaran, Umadevi Subramanian, Bharanidharan Devarajan

**Affiliations:** 1 Department of Bioinformatics, Aravind Medical Research Foundation, Madurai, Tamil Nadu 625020 India; 2 School of Chemical and Biotechnology, SASTRA (Deemed to be University), Thanjavur, Tamil Nadu 613401 India

**Keywords:** Whole exome sequencing, Simulated exome data, Human reference genome, Variant calling pipelines, SNVs and InDels

## Abstract

**Background:**

Whole exome sequencing (WES) is a cost-effective method that identifies clinical variants but it demands accurate variant caller tools. Currently available tools have variable accuracy in predicting specific clinical variants. But it may be possible to find the best combination of aligner-variant caller tools for detecting accurate single nucleotide variants (SNVs) and small insertion and deletion (InDels) separately. Moreover, many important aspects of InDel detection are overlooked while comparing the performance of tools, particularly its base pair length.

**Results:**

We assessed the performance of variant calling pipelines using the combinations of four variant callers and five aligners on human NA12878 and simulated exome data. We used high confidence variant calls from Genome in a Bottle (GiaB) consortium for validation, and GRCh37 and GRCh38 as the human reference genome. Based on the performance metrics, both BWA and Novoalign aligners performed better with DeepVariant and SAMtools callers for detecting SNVs, and with DeepVariant and GATK for InDels. Furthermore, we obtained similar results on human NA24385 and NA24631 exome data from GiaB.

**Conclusion:**

In this study, DeepVariant with BWA and Novoalign performed best for detecting accurate SNVs and InDels. The accuracy of variant calling was improved by merging the top performing pipelines. The results of our study provide useful recommendations for analysis of WES data in clinical genomics.

**Electronic supplementary material:**

The online version of this article (10.1186/s12859-019-2928-9) contains supplementary material, which is available to authorized users.

## Background

Whole genome sequencing (WGS) and Whole exome sequencing (WES) methods are applied in clinical settings for detecting patient’s genomic variants and etiology of the disease. Whole exome sequencing (WES), is becoming a standard, more economic approach to genome sequencing [1]. Although it covers only exonic regions (< 2% of the whole genome), it produces a large quantity of data (raw reads) that requires a significant amount of bioinformatics analysis to create biologically meaningful information [2].

WES output must be accurate and consistent in detecting specific variants that impact a particular phenotype. The first obstacle to accurate variant detection is the technical error when exome capturing kits do not capture the regions of interest which increases the possibility of missing some potential variants [3]. Secondly, variants detection may be missed by the variant calling pipelines. Though many variant callers are available [4, 5], each performs best with the data obtained from a particular sequencing platform. For example, SAMtools is best for Ion Proton data [6], and GATK is best for Illumina data [7]. They have also shown low concordance when examining the same set of sequencing data. Thus the accuracy of the variant callers is still not adequate [8, 9].

No single pipeline with the combination of aligner and variant caller has demonstrated superiority in detecting all the variants. Applying multiple tools can result in more misleading output [10]. It has also been reported that read aligners influence the accuracy of variant detection [9, 11]. Thus, it is essential to evaluate variant calling pipelines with the optimal combination of aligners and variant callers that may produce accurate variant calls including single nucleotide variants (SNVs) and small insertion and deletion (InDels).

Several benchmarking studies have been conducted to assess the performance of different variant calling pipelines in detecting accurate variants. Liu et al. compared the performance of four variant callers using single and multi-sample variant-calling strategies. They reported that GATK performed best on real and simulated exome data, while SAMtools could be used to detect higher true positive SNVs on simulated whole genome sequencing data [12]. In another study, based on the read-depth, allele balance and mapping quality, GATK outperformed SAMtools on low coverage exome data [13]. In a separate study, Roberts et al. used cancer-normal exome sequencing data in detecting only SNVs*.* They reported a substantial difference in detecting SNVs by different algorithms with respect to the number and the character of sites [14]. Many benchmarking studies use the set of NA12878 Genome in a Bottle (GiaB) high confidence GRCh37 variants as a gold standard reference set [6, 8, 11, 15, 16]. However, several questions remain to be answered about how different pipelines perform with the improved version of the human reference genome GRCh38 and how the newly developed tools perform.

The accurate detection of InDels is more challenging than SNVs because of the limited guidelines [17, 18]. The issues in InDel detection are low concordance rate among different sequencing platforms, realignment error, error near perfect repeat regions and incomplete reference genome in some cases [19, 20]. Even though recent advancements in NGS have improved the sensitivity of different sequencing platforms, enhancing InDel calling accuracy is still a significant issue [21]. In order to identify the most accurate InDel calling tools, recent studies have attempted to evaluate these tools focusing only on InDel calling. One comparative study of four variant callers using the human exome data reported that GATK had a high sensitivity for InDel detection. The study further indicated that most InDels called by variant callers were < 10 bp in length and that the performance of four algorithms was unaffected by InDel size [21]. However, another comparative study of seven InDel callers using 78 human genome data indicated that performance differed depending on the number and size [18]. Similarly, based on the simulated data, Neuman et al. reported a discrepancy in InDel calling efficiency at higher InDel size [22]. Other studies noted that the detection of large sized InDels is more difficult than identification of small InDels [19, 23]. Yet, despite the existence of many tools for InDel detection, a study focusing on the evaluation of current tools with respect to various performance metrics is sparse.

In the present study, we sought to assess the best combination of aligner with variant caller tools for detecting SNVs and InDels separately. To achieve this aim, we used the real whole human exome sequencing dataset NA12878 and the simulated exome data. Additionally, we compared the performance of pipelines using two new exome data sets NA24385 and NA24632 available from GiaB consortium. We report here several performance metrics with respect to F-score aimed to build an extensive benchmark study to asses the performance of pipelines with currently available well-known tools, used for detecting SNVs and InDels.

## Results

In order to assess the performance of variant calling pipelines in terms of their capacity to accurately detect SNVs and InDels from WES datasets, we developed 20 pipelines with the combinations of four variant caller and five aligner tools. The results were validated with high confidence truth set from GiaB for both the human GRCh37 and GRCh38 reference and compared the results with gold standard truth variants provided by GiaB consortium.

### Performance of variant calling pipelines

**Fig. 1:**
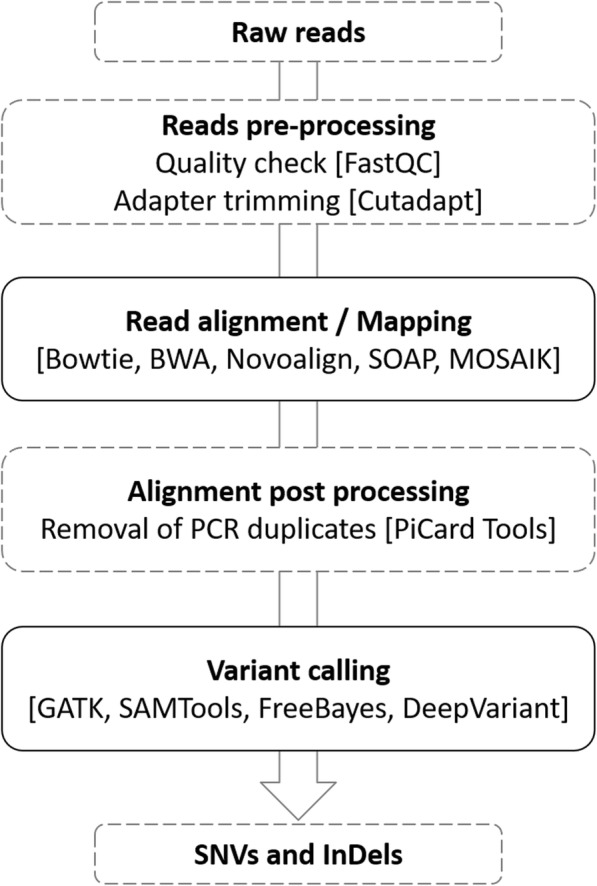
Schematic of the NGS data analysis pipeline

Initially, we checked the quality of human exome dataset NA12878 and trimmed the adapter sequence. Then, we used five different aligners to map the reads with the reference genomes GRCh37 and GRCh38, as shown in Fig. 1 (further details are given in Additional file 6: Table S1). After the post-alignment process, we used four different variant calling tools, namely GATK, SAMtools, FreeBayes, and DeepVariant. Next, using 20 different pipelines, SNVs were detected in four exome datasets (i) NA12878 aligned with GRCh38 genome (exome-1), (ii) simulated exome using GRCh38 genome (exome-2), (iii) NA12878 aligned with GRCh37 genome (exome-3), and (iv) simulated exome using GRCh37 genome (exome-4). We ran the pipelines on our server (340 GB RAM with 40 core for exome-1 and exome-2 and 320 GB RAM with 32 core for exome-3 and exome-4; and the run time for each pipeline was showed in Additional file 7: Table S2). To assess the performance of pipelines, we calculated true positive (TP), false positive (FP) and false negative (FN) variants using GiaB truth set, as it contains 23,686 SNVs and 1258 InDels for NA12878 exome. We used F-score as the measure of performance quality.

**Fig. 2:**
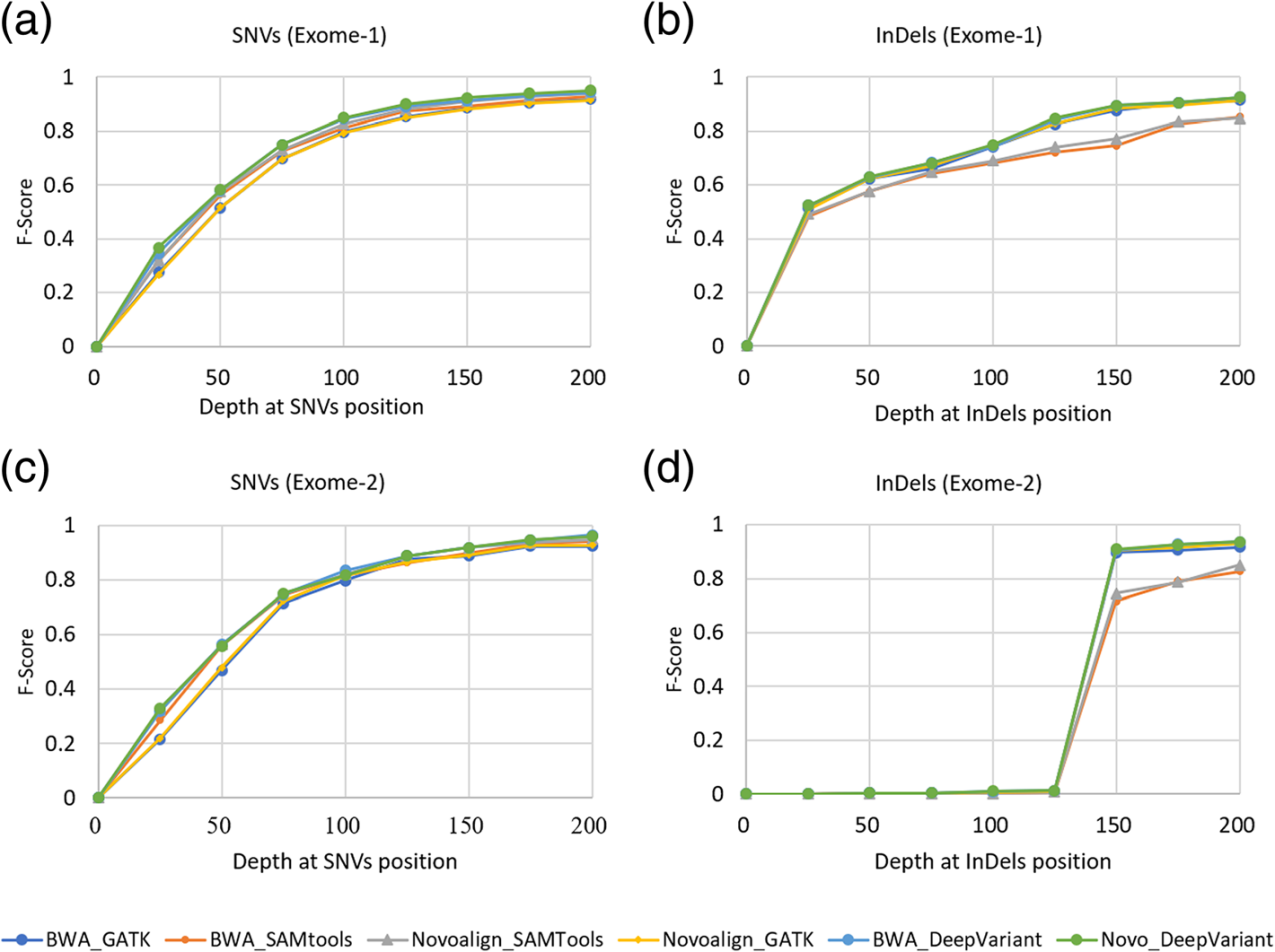
F-score with respect to depth of coverage for top six pipelines. ROC curves were plotted using F-score of each pipeline against the depth at the SNVs (**a**, **c**) and InDels (**b**, **d**) positions on exome-1 (**a**, **b**) and exome-2 (**c**, **d**)

In all the exome datasets, BWA_DeepVariant, Novoalign_DeepVariant, BWA_SAMtools and Novoalign_SAMtools were the top performing pipelines for the SNVs (Additional file 8: Table S3). The F-scores of these four pipelines were 0.97 on exome-1; 0.99 (except BWA_SAMtools) on exome-2; 0.98 (except BWA_SAMtools) on exome-3; and 0.98 on exome-4. In the case of InDels, BWA_DeepVariant and Novoalign_DeepVariant performed best followed by BWA_GATK and Novoalign_GATK. Moreover, DeepVariant based pipelines performed better than those based on GATK, which showed the highest F-score of 0.99 on all exomes (Additional file 8: Table S3). Further, to explore how the sequencing depth affects the performance, we plotted the receiver operating characteristic (ROC) curves, representing F-score of top six performing pipelines as a function of the depth detected at variant positions on all exomes (Fig. 2 and Additional file 1:Figure S1). The top three pipelines showed a similar profile for both SNVs and InDels. The next three pipelines performed roughly at the same level for SNVs; while they showed a subtle difference in performance for InDels only at 175X depth of coverage (Fig. 2d and Additional file 1: Figure S1d). Most of the SNVs and InDels were detected at about 150X depth of coverage, suggesting that this depth is a sufficient parameter for detecting the variants.

**Fig. 3:**
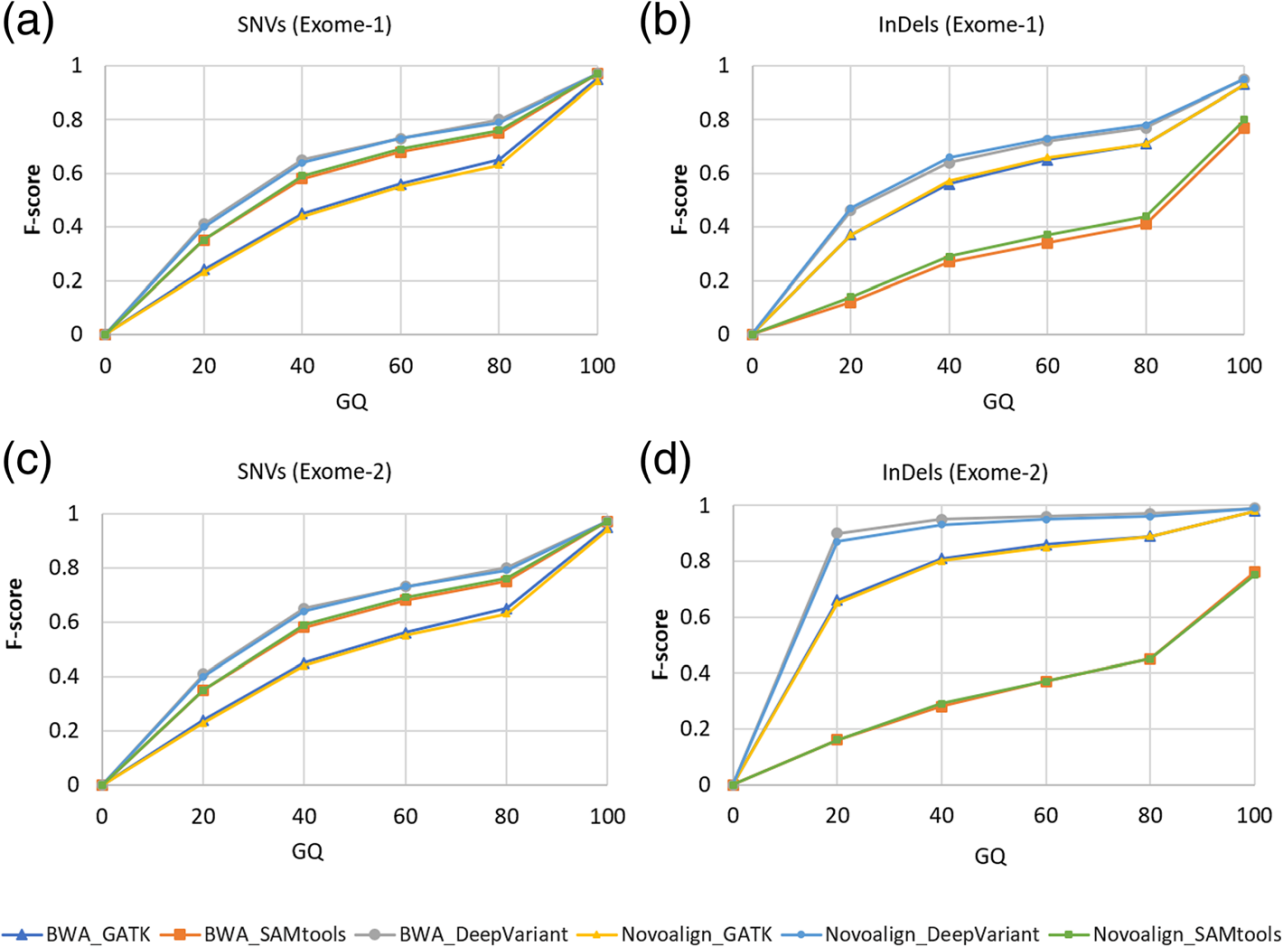
F-score with respect to genotype quality (GQ) for top six pipelines. ROC curves were plotted using the GQ of SNVs (a, c) and InDels (**b**, **d**) against F-score using exome-1 (**a**, **b**) and exome-2 (**c**, **d**)

Next, we assessed the performance of each pipeline using F-score with respect to genotype quality (GQ). At GQ > 60, all the top six pipelines showed better performance for both SNVs and InDels on all four exomes (Additional file 9: Table S4). BWA_DeepVariant and Novoalign_DeepVariant performed the best among the six pipelines for both SNVs and InDels on all exomes (Fig. 3 and Additional file 2: Figure S2), followed by BWA_SAMtools and Novoalign_SAMtools in case of SNVs; and Novoalign_GATK and BWA_GATK in case of InDels. We observed that the performance of pipelines increased along with the increased GQ value. However, BWA_DeepVariant and Novoalign_DeepVariant performed well, even at low GQ values on simulated exome data (Fig. 3d and Additional file 2: Figure S2).

To further evaluate the pipelines, we used F-score with respect to genotype concordance. We observed that the top six pipelines performed comparably well as observed using Depth of coverage and GQ metrics (Additional file 10: Table S5). We also investigated the ratio of heterozygous to homozygous (het/hom) and found that the ratio for detecting SNVs was higher than InDels. The ratio for SNVs was ~ 1.6 on exome-1 and exome-2; ~ 1.5 on exome-3 and exome-4. While the ratio for InDels was ~ 1.2 for exome-1 and exome-2; ~ 1.2 on exome-3; and ~ 1.3 on exome-4. Indeed, we observed the difference in the performance of pipelines when we compared the heterozygous and homozygous detection with respect to F-score (Additional file 11: Table S6). Based on this F-score, BWA_DeepVariant, Novoalign_DeepVariant, BWA_SAMtools, and Novoalign_SAMtools (F-score > 0.96) performed comparably well in detecting SNVs on all exomes. While BWA_DeepVariant, Novoalign_DeepVariant, BWA_GATK and Novoalign_GATK performed well (F-score > 0.9) in detecting InDels.

### Performance in detecting SNVs using Ti/Tv ratio

We calculated the ratio of transition (Ti) to transversion (Tv), one of the key quality metrics in detecting SNVs. The Ti/Tv ratio was ~ 3.4 on exome-1 and exome-2, and ~ 3.2 on exome-3 and exome-4. Indeed, we also investigated F-score with respect to transition (Ti) and transversion (Tv) compared to gold standards. Based on the F-score, Novoalign_DeepVariant and BWA_DeepVariant performed best on all exomes followed by Novoalign_SAMtools and BWA_SAMtools (Additional file 12: Table S7).

### Performance in detecting InDel at different base pair (bp) length

**Fig. 4:**
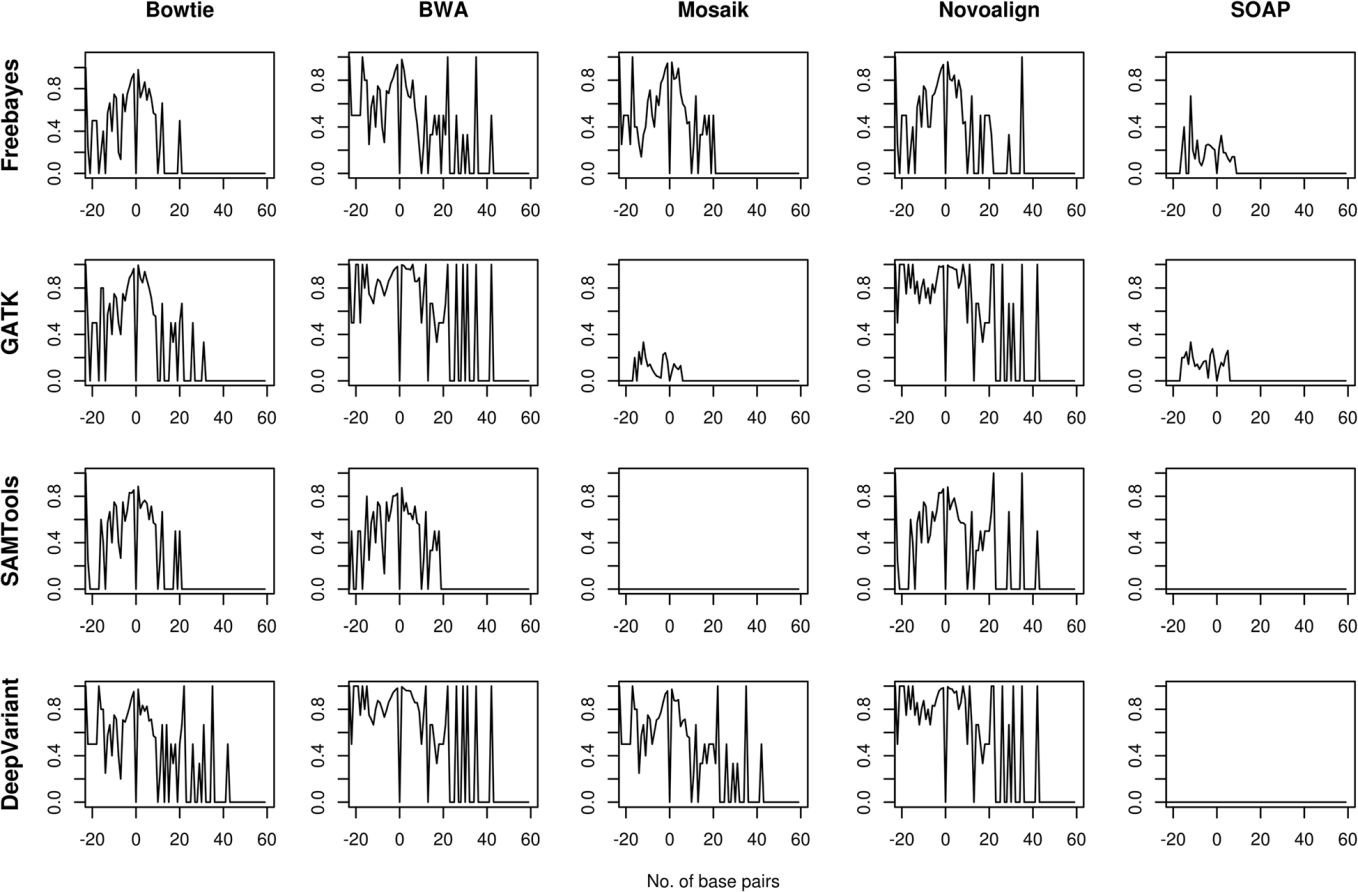
The InDels detection performance of pipelines on exome-1. F-scores of InDels were plotted against the base pair length of the InDels. The negative value of x-axis indicates the deletion and positive value for insertion

**Fig. 5:**
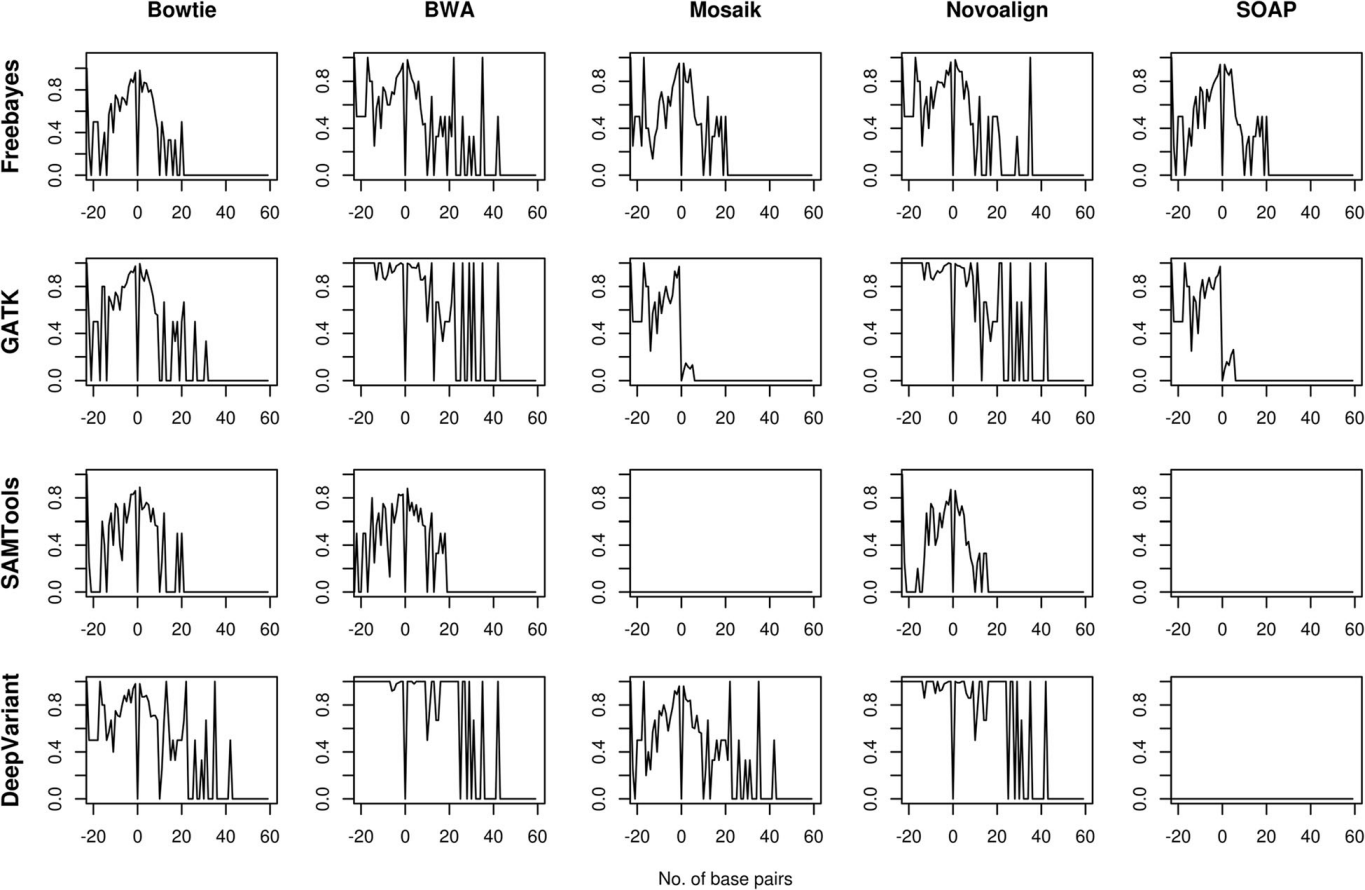
The InDels detection performance of pipelines on exome-2. F-scores of InDels were plotted against the base pair length of the InDels. The negative value of x-axis indicates the deletion and positive value for insertion

We analyzed the InDel detection performance of the pipelines using F-score with respect to base pair length of insertion and deletion. DeepVariant and GATK pipelines, along with the aligners BWA and Novoalign, performed comparably well at higher base pair length on all the exomes. However, the performance of each pipeline differed at particular bp length of InDels. Mostly, the pipelines performed better at 17, 23, 25 and 26 bp length deletions; and at 22 and 35 bp length insertions (Figs. 4, 5 and Additional file 3: Figure S3 and Additional file 4: Figure S4). BWA_DeepVariant and Novoalign_DeepVariant performed best in terms of detecting the number of insertions on exome-2 and exome-4. All pipelines failed to detect the deletions with 24 and 27 bp length on exome-1, exome-3 and exome-4, and insertions with 13, 23, 24 and 27 bp length on exome-1 and exome-3; 59 bp length on exome-1, exome-2 and exome-3 (Additional file 13: Table S8). We pointed out the possible reasons for the failure in detecting InDels at particular base pair length in the discussion section.

### Comparison of best-performing pipelines

**Fig. 6:**
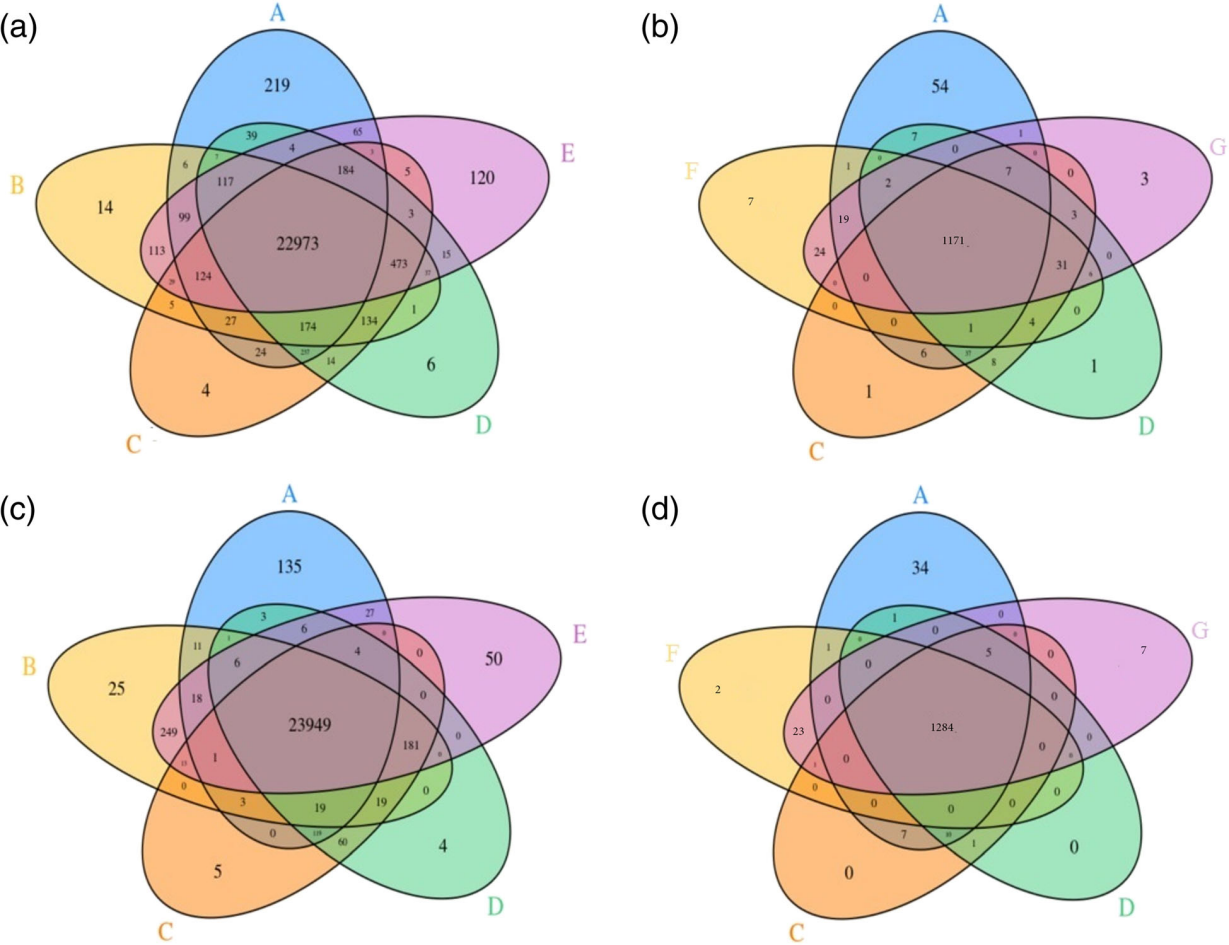
Venn diagram depicting the comparison of top four pipelines. GiaB variants (**a**) compared against the top 4 performing pipelines (**b**) BWA_SAMtools, (**c**) BWA_DeepVariant, (**d**) Novoalign_DeepVariant, (**e**) Novoalign_SAMtools, (**f**) BWA_GATK and (**g**) Novoalign_GATK for SNVs (**a**, **c**) and InDels (**b**, **d**) on exome-1 (top row) and exome-2 (bottom row)

In order to improve the accuracy in detecting variants, we compared the GiaB truth variants against the specific variants detected by the top four pipelines (mentioned earlier). We compared BWA_DeepVariant, Novoalign_DeepVariant, BWA_SAMtools, and Novoalign_SAMtools for SNVs (Fig. 6a, c), and BWA_GATK, Novoalign_GATK, BWA_DeepVariant, and Novoalign_DeepVariant for InDels detection (Fig. 6b, d) on exome-1 and exome-2. We illustrated a similar analysis of comparison on exome-3 and exome-4 in Additional file 5: Figure S5.

We observed high concordance of the variants with GiaB truth set by merging four pipelines (Figures 6 and Additional file 5: Figure S5). We showed that the accuracy in detecting true positive (TP) SNVs improved to ~ 99% on exome-1 and exome-2, and ~ 98% on exome-3 and exome-4. We also observed ~ 96% on exome-1 and exome-3, and ~ 98% on exome-2 and exome-4 (simulated exomes) for InDels. Further, we investigated the performance improvement by merging the top two variant calling pipelines DeepVariant_BWA and DeepVariant_Novoalign, which improved TP detection to ~ 98% and ~ 96% for SNVs and InDels respectively on all the exomes. Our results showed that merged pipelines performed better than the independent pipelines; despite the increased FDR.

**Fig. 7:**
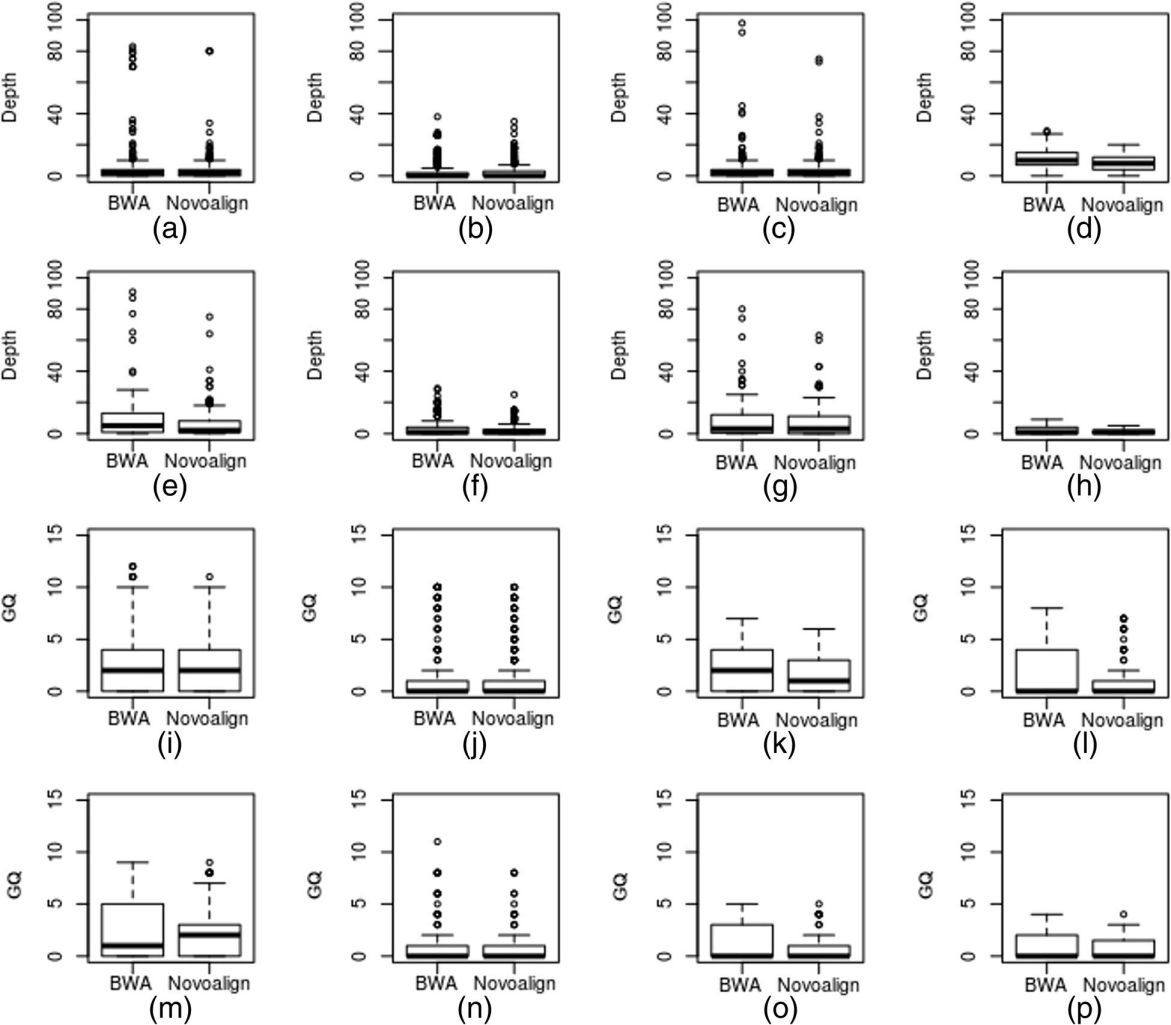
Analysis of depth and GQ of true SNVs missed (FN) by BWA and Novoalign alignments. Depth of the false negative SNVs on exome-1(**a**), − 2 (**b**), − 3(**c**) and − 4 (**d**) and InDels on exome-1(**e**), − 2(**f**), − 3(**g**) and − 4(**h**). Genotype quality of false negative SNVs on exome-1 to - 4 (**i**, **j**, **k**, and **l**) and InDels on exome-1 to − 4 (**m**, **n**, **o** and **p**) respectively

Even though each variant caller uses different algorithms (strategy to identify the variants as given in Additional file 6: Table S1), we observed ~ 0.5–1.5% and ~ 0.5–4% false negative (FN) SNVs and InDels respectively on all the exomes. To investigate further, we plotted depth and the genotype quality (GQ) of the FN variants obtained by BWA and Novoalign alignments (Fig. 7). We observed that they all fell under the upper limit of 30X depth on all the exomes (Fig. 7a-h). However, the presence of outliers suggesting that the depth might not be the only reason for the true missing variants (FNs). Based on the GQ analysis (Fig. 7i-p), we observed that all FNs had < 10 GQ on all the exomes, which suggested that variant callers possibly missed the true variants due to the low GQ.

### Performance comparison of variant calling pipelines using NA24385 and NA24631 datasets

**Fig. 8:**
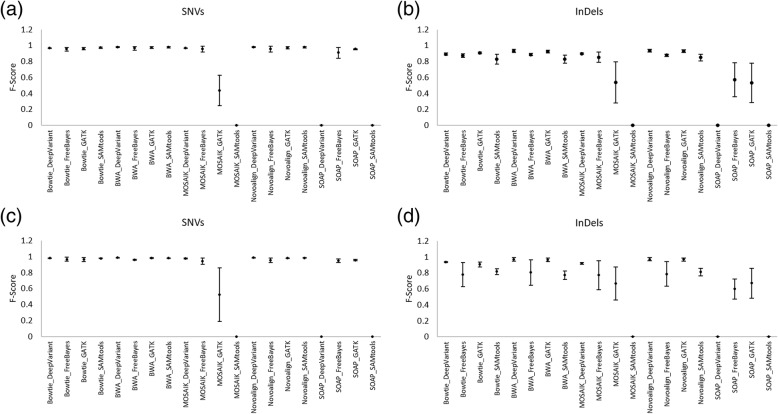
Performance comparison of pipelines using F-score on NA12878, NA24385, and NA24631. The values and the error bars represent the average and standard deviation of F-score respectively, obtained from all three datasets. Performance comparison of pipelines in detecting SNVs GRCh38 (**a**, **b**) and InDels GRCh37 (**c**, **d**)

In addition to NA12878, we assessed the performance of variant calling pipelines using two human whole exome datasets NA24385 and NA24631. By comparing the average and standard deviation of F-score of three human exome data sets for each pipeline, we observed no significant change in the top performing pipelines (Fig. 8). Indeed, we observed that DeepVariant with the aligners BWA and Novoalign performed best invariably with all data sets (Fig. 8 and Additional file 14: Table S9).

## Discussion

A major challenge in whole exome sequencing (WES) is how to process the data to detect accurate variants that cause the disease. This process requires an alignment and variant calling tool. Since many aligner and variant caller tools are available, in this study, we have compared 20 pipelines that consist of a combination between five popular aligners and four popular variant callers. We have used the human exome NA12878, which has a high confidence truth variant set, for assessing the performance of each pipeline. We have also used simulated exome data, which is being most popular for evaluating biological models or understanding about specific datasets [24]. Both real and simulated data are necessary to compare the results as they provide different assessment strategies. Our results show that the overall performance of each pipeline is similar in real and simulated exome data. However, the false discovery rate (FDR) is much lesser in simulated than real data, which could be due to the underlying error model of experimental exome sequencing.

Although we use F-score with respect to several performance metrics, the Ti/Tv ratio is one of the key performance metrics in detecting SNVs. Therefore, we first calculated the ratio to assess performance for SNVs detection. We have indicated that the ratio is transiently following the reported range of 2.6–3.3 [25] on all exomes; except SOAP_GATK on exome-3 (3.55) and Mosaik_GATK on exome-4 (3.94). However, the Ti/Tv ratio may not always necessarily mean an accurate performance metric because low-frequency SNVs sometimes have a higher ratio than the moderate-frequency SNVs [7]. In this study, as reported by McKenna et al. [26], we have observed the higher ratio with the accurate variant set. Therefore, we have used the Ti/Tv ratio to examine the accuracy in detecting true positive SNVs and F-score to assess the overall pipeline performance. Our results [Additional file 15: Table S10] along with the previous report by Hwang et al. [6] highlight that SAMtools outperforms GATK in detecting SNVs; in contrast to other reports [12, 13]. However, DeepVariant performed best among the variant callers.

The overall performance of pipelines in detecting InDels is comparatively lower than SNVs detection. This low performance could be due to WES data as they miss many large InDels [19]. Further, we have compared our results with previous benchmarking studies that used GiaB gold standard variant dataset NA12878 (Additional file 15: Table S10) [6, 8, 11, 15, 16]. Our results show that DeepVariant outperformed all the variant callers in contrast to previous studies that GATK consistently performed well for InDel detection. Moreover, DeepVariant has detected more InDels at higher base pair length size than GATK.

Further, we have investigated the influence of aligners, particularly BWA and Novoalign (non-commercial version). The algorithm of BWA balances between running time, memory usage, and accuracy, while Novoalign shows slow and high memory usage that contribute to better mapping. BWA performs better for SNVs and Novoalign for InDels using NA12878 in an agreement with previous reports (Additional file 15: Table S10). Also both aligners perform equally well with subtle differences using NA24385 and NA24631. However, our results indicate that variant caller has more influence in detecting SNVs and InDels than the aligners.

Finally, the selection of the human reference genome is a prerequisite for successful analysis of WES; we have conducted the analysis comparing SNVs and InDels detected based on GRCh38 and GRCh37. Our results show that the pipelines perform slightly better with GRCh38 than GRCh37, possibly due to more true positive (TP) SNVs and InDels detected. In case of missing variants (FNs), GRCh38 has lower (~ 8%) and much lower (~ 20%) number of FNs for SNVs and InDels respectively than GRCh37. Furthermore, our investigation on FNs has indicated that show genotype quality and depth of the coverage influence the FN detection (Fig. 7). In this study, we report GRCh38 is preferred genome for evaluation studies. Moreover, it is reported to offer high coverage, more accurate genomic analysis and improved annotation of the centromere regions [27].

## Conclusions

In this study, we demonstrated that the variant caller DeepVariant in combination with aligner BWA or Novoalign perform best in detecting accurate SNVs and InDels. Furthermore, we recommend that merging of BWA and Novoalign aligners with DeepVariant and SAMtools callers improve accuracy for SNVs detection; and with DeepVariant and GATK for InDels detection. However, the users should be aware that the pipelines may fail to detect ~ 1% to ~ 2% of true variants. To conclude, our benchmarking analysis can assist the investigators in choosing a variant calling pipeline for accurate detection of SNVs and InDels, and will greatly aid disease-causing variants detection from WES data.

## Methods

### Datasets

FASTQ files of human exome HapMap/1000 CEU female NA12878 (accession No.: SRR098401) was downloaded from NCBI-Sequence Read Archive (SRA- https://www.ncbi.nlm.nih.gov/sra). The whole exome sequencing of NA12878 was performed using the HiSeq Illumina 2000 platform and SureSelect human all exon v2 target capture kit [28]. The target region BED file was downloaded from Agilent SureDesgin (https://earray.chem.agilent.com/suredesign/, ELID: S0293689). The human reference genomes GRCh37 and GRCh38 were downloaded from the Ensembl [29]. Next, NA12878 high confidence call set version 2.19 by Genome in a Bottle (GiaB) consortium was used for pipeline performance validation. The variant set along with a BED file was downloaded from NCBI and was further filtered to highly accurate call set using the BED file. This GiaB variant set, created by integrating 14 different datasets from five sequencers, is the only ‘gold standard’ variant dataset publically available for systematic comparison of variant callers. Furthermore, two recently released datasets NA24385 (Ashkenazim male; accession No.: SRR2962669) and NA24631 (Chinese male; SRR2962693) from GiaB were downloaded for the comparison. These datasets were generated using Agilent SureSelect Human All Exon v5 kit for capturing and HiSeq Illumina 2500 platform for sequencing.

Further, to test the certainty of the performance of the pipelines, simulated human whole exome data was generated by ART toolkit [30]. ART takes a reference genome in FASTA format and generates ‘synthetic’ sequencing reads. The reference genomes GRCh37, GRCh38, and sequencing target BED (SureSelect human all exon v2 target capture region) file were inputs of the simulator. The simulated short paired-end reads were generated with parameters of 150 bp length; the depth of 150X covering sequencing targets; and Illumina HiSeq 2000 sequencing technology with 0.01% error model. This simulated exome data mimic the technology-specific sequencing process with customized read length and error characteristics.

### Pipeline development

We developed the modular pipeline (Fig. 1) that consist of the aligner and variant caller tool, to analyze both the real and simulated exome data sets. The pipeline involves several steps to produce high-quality alignment files and to predict particular variants. Initially, the quality of the raw reads obtained from SRA was checked by FastQC [31], and the low-quality reads and adapter sequences were removed by Cutadapt [32]. Next, high-quality reads were aligned with the human reference genome GRCh37 and GRCh38. After the alignment, PCR duplicates were removed using PiCard Tools [33]. Finally, SNVs and InDels were detected using different variant calling tools. Based on prevalence and popularity, five aligners and four variant callers were used in combination to develop 20 different pipelines (Additional file 6: Table S1). The pipeline was written using UNIX shell script with default parameters (available on https://github.com/bharani-lab/WES-pipelines.git).

### Performance evaluation of variant calling pipelines

The variants determined by pipelines were compared with standard variants provided by GiaB using VCFtools [34]. The SureSelect Human All Exon v2 target captured kit bed file (https://earray.chem.agilent.com/suredesign/, ELID: S0293689) was used to capture the locations of variants. Tabix was used to extract the variants using this target capture bed file, and vcflib tool *vcfallelicprimitives* was used to pre-process the vcf files. The variant calling pipeline performance was measured statistically as sensitivity = TP / (TP + FN), precision = TP / (TP + FP), false discovery rate (FDR) = FP / (TP + FP) and F-score = 2TP / (2TP + FP + FN). TP is a true positive variant that exists in GiaB data set and also is detected by the pipeline; FP is a false positive variant that does not exist in GiaB and is detected by the pipeline; FN is a false negative variant that exists in GiaB and is not detected by the pipeline. F-score was used as the key metric for evaluating the performance of the pipelines.

Furthermore, F-score with respect to depth of coverage, heterozygous (Het) and homozygous (Hom) detection, transition (Ti) and transversion (Tv) conversion of SNVs, genotype quality, genotype concordance, insertion and deletion size were calculated for the pipeline performance evaluation. Depth of coverage, which is the total number of bases sequenced and aligned at a given reference base position, was calculated by the GATK package *DepthOfCoverage*. The metrics Het/Hom and Ti/Tv ratios were calculated as described by Wang et al. [35]. The genotype quality is used to estimate the accuracy of a genotype call and is defined by GQ = − 10 * log_10_(Error rate). The genotype (allele) concordance, which is the intersection of the ‘test’ and ‘truth’ datasets, was determined by *Concordance* package of SnpSift. Venn diagram was plotted to compare the performance of top performing pipelines.

## Additional files

Additional file 1: **Figure S1.** F-score with respect to depth of coverage for top six pipelines. ROC curves were plotted using the depth of SNVs (a, c) and InDels (b, d) against F-score using exome-3 (a, b) and exome-4 (c, d). (PNG 148 kb)

Additional file 2: **Figure S2.** F-score with respect to genotype quality for top six pipelines. ROC curves were plotted using the GQ of SNVs (a, c) and InDels (b, d) against F-score using exome-3 (a, b) and exome-4 (c, d). (PNG 181 kb)

Additional file 3: **Figure S3.** InDels detection performance in exome-3. F-scores of InDels were plotted against the base pair length of the InDels. The negative value of x-axis indicates the deletion and positive value for insertion. (PNG 1076 kb)

Additional file 4: **Figure S4.** InDels detection performance on exome-4. F-scores of InDels were plotted against the base pair length of the InDels. The negative value of x-axis indicates the deletion and positive value for insertion. (PNG 915 kb)

Additional file 5: **Figure S5.** Venn diagram depicting the comparison of top four pipelines. GiaB variants (A) compared against the top performing pipelines (B) BWA_SAMtools, (C) BWA_DeepVariant, (D) Novoalign_DeepVariant, (E) Novoalign_SAMtools, (F) BWA_GATK and (G) Novoalign_GATK for SNVs (a, c) and InDels (b, d) on exome-3 (top row) and exome-4 (bottom row). (PNG 2321 kb)

Additional file 6: **Table S1.** Tools used in pipeline development. (PDF 217 kb)

Additional file 7: **Table S2.** Run time (in min) of 20 variant calling pipelines. (PDF 186 kb)

Additional file 8: **Table S3.** Performance of twenty pipelines. Performance of pipelines analyzed for SNVs and InDels on real (exome-1 and exome-3) and simulated exome data (exome-2 and exome-4). (PDF 386 kb)

Additional file 9: **Table S4.** Performance (F-score) of pipelines with respect to genotype quality (GQ) for SNVs and InDels (PDF 383 kb)

Additional file 10: **Table S5.** F-score of pipelines for SNVs and InDels. F-score was used as the function of genotype concordance (PDF 139 kb)

Additional file 11: **Table S6.** F-score of pipelines for heterozygous and homozygous variants, and Heterozygous/Homozygous ratio. (PDF 290 kb)

Additional file 12: **Table S7.** F-score of pipelines for transition and transversion detection, and Ti/Tv ratio. (PDF 215 kb)

Additional file 13: **Table S8.** Number of deletions and insertions with different base pair lengths detected by pipelines. (XLSX 59 kb)

Additional file 14: **Table S9.** Performance of 20 pipelines on NA24385 and NA24631 data sets using both reference genomes GRCh38 and GRCh37 for SNVs and InDels. (PDF 420 kb)

Additional file 15: **Table S10.** Comparison of three benchmarking studies that used GiaB gold standard variant dataset NA12878 (PDF 202 kb)

## Data Availability

The data that support the findings of this study are openly available in SRA-NCBI. Database. These data set can be downloaded from the following resources available in the public domain: NA12878 (https://www.ncbi.nlm.nih.gov/sra/?term=SRR098401). NA24385 (https://www.ncbi.nlm.nih.gov/sra/?term=SRR2962669). NA24631 (https://www.ncbi.nlm.nih.gov/sra/?term=SRR2962693). GiaB (https://ftp-trace.ncbi.nlm.nih.gov/giab/ftp/data/). The simulated data used in this study can be generated and a detailed tutorial is openly. available at GitHub (https://github.com/bharani-lab/WES-pipelines.git).

## Abbreviations

FDR: False Discovery Rate
FN: False Negative
FP: False Positive
GiaB: Genome in a Bottle
GQ: Genotype Quality
InDels: Insertions and Deletions
SNVs: Single Nucleotide Variants
Ti: Transition
TP: True Positive
Tv: Transversion
WES: Whole Exome Sequencing
WGS: Whole Genome Sequencing
