# Supplementary figures and images for: Performance assessment of variant calling pipelines using human whole exome sequencing and simulated data
Source: BMC Bioinformatics. 2019 Jun 17;20:342. doi: 10.1186/s12859-019-2928-9 (PMC6580603; doi:10.1186/s12859-019-2928-9)

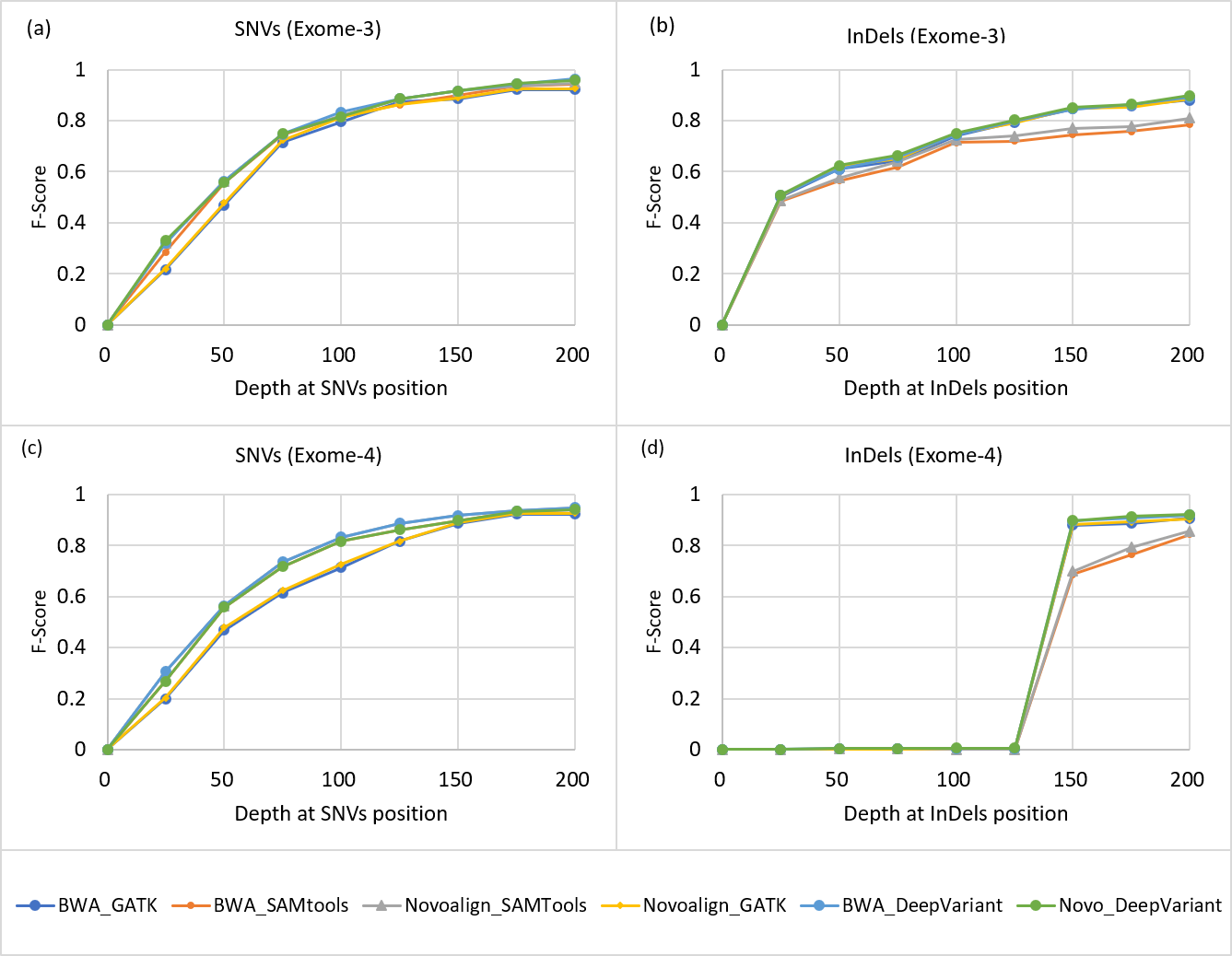

Supplement: Supplementary file 1 — Figure S1. F-score with respect to depth of coverage for top six pipelines. ROC curves were plotted using the depth of SNVs (a, c) and InDels (b, d) against F-score using exome-3 (a, b) and exome-4 (c, d). (PNG 148 kb) [file 12859_2019_2928_MOESM1_ESM.png]

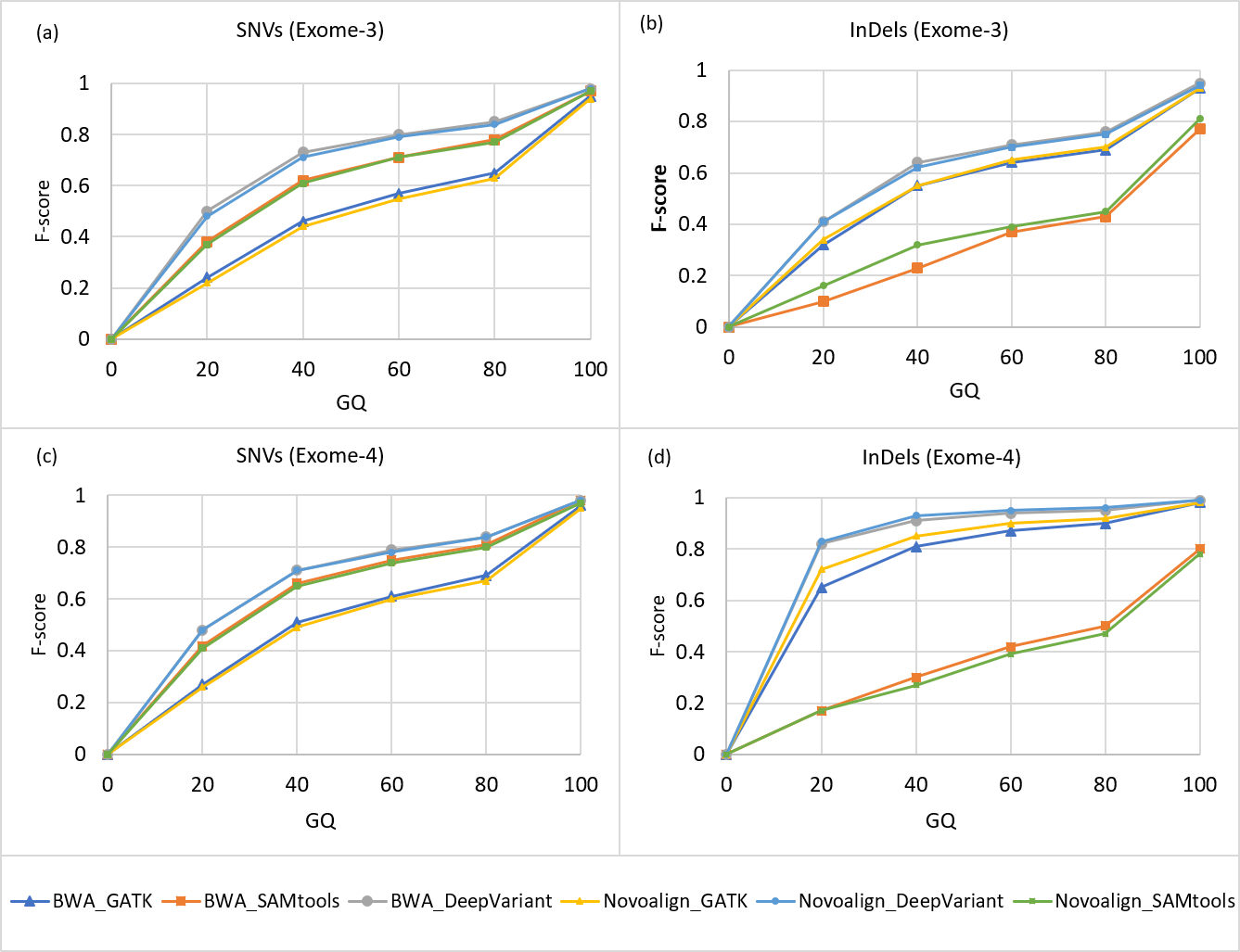

Supplement: Supplementary file 2 — Figure S2. F-score with respect to genotype quality for top six pipelines. ROC curves were plotted using the GQ of SNVs (a, c) and InDels (b, d) against F-score using exome-3 (a, b) and exome-4 (c, d). (PNG 181 kb) [file 12859_2019_2928_MOESM2_ESM.png]

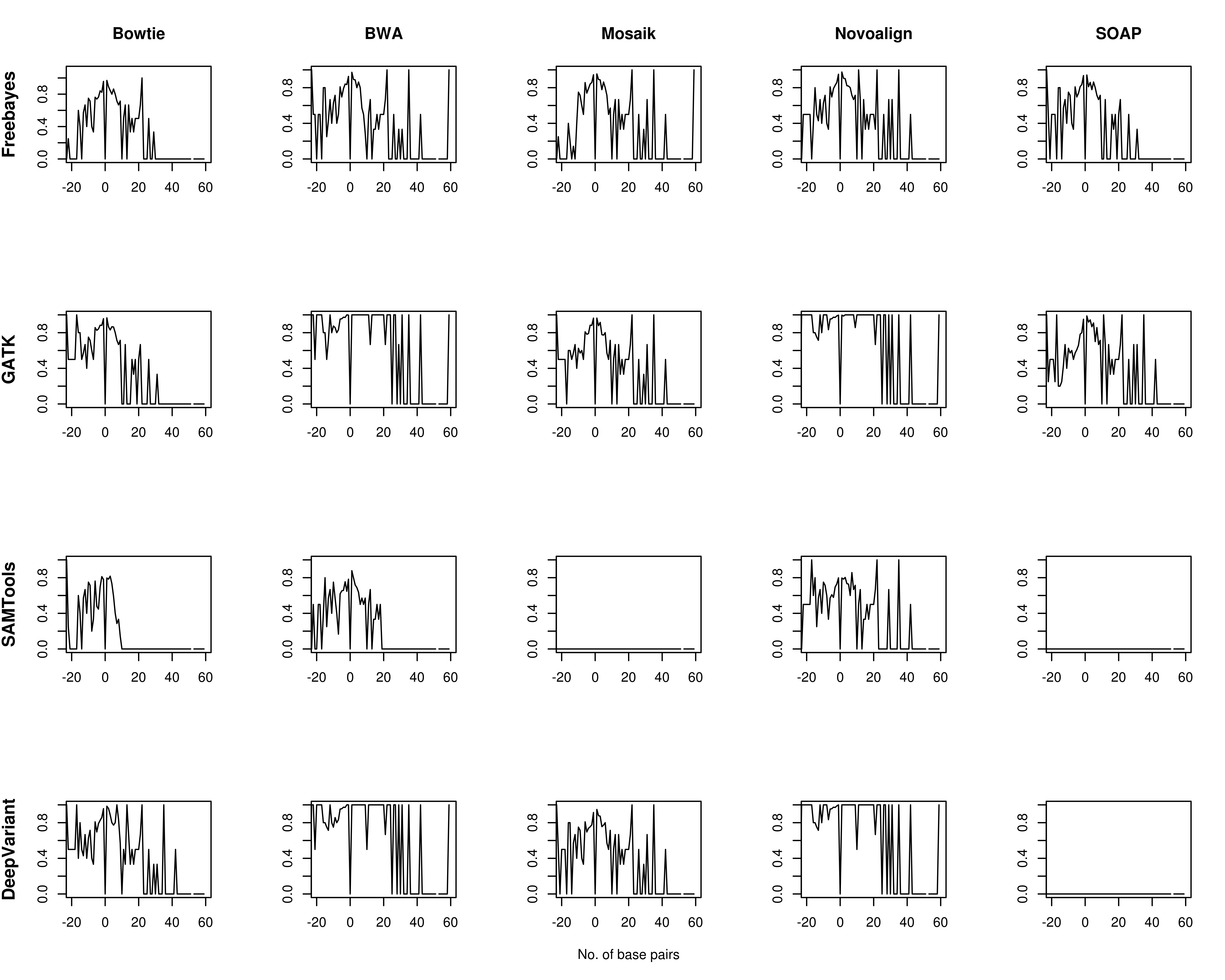

Supplement: Supplementary file 3 — Figure S3. InDels detection performance in exome-3. F-scores of InDels were plotted against the base pair length of the InDels. The negative value of x-axis indicates the deletion and positive value for insertion. (PNG 1076 kb) [file 12859_2019_2928_MOESM3_ESM.png]

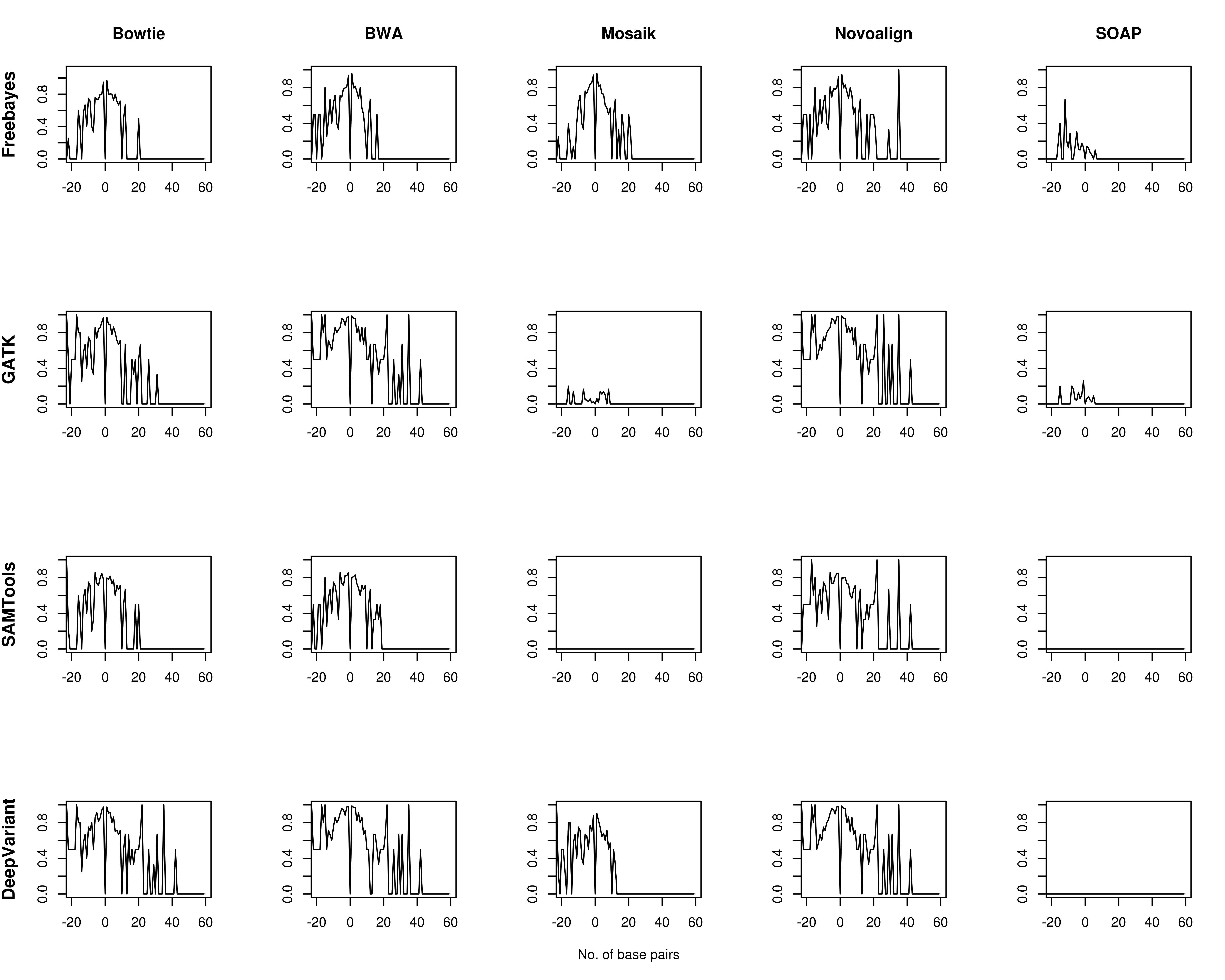

Supplement: Supplementary file 4 — Figure S4. InDels detection performance on exome-4. F-scores of InDels were plotted against the base pair length of the InDels. The negative value of x-axis indicates the deletion and positive value for insertion. (PNG 915 kb) [file 12859_2019_2928_MOESM4_ESM.png]

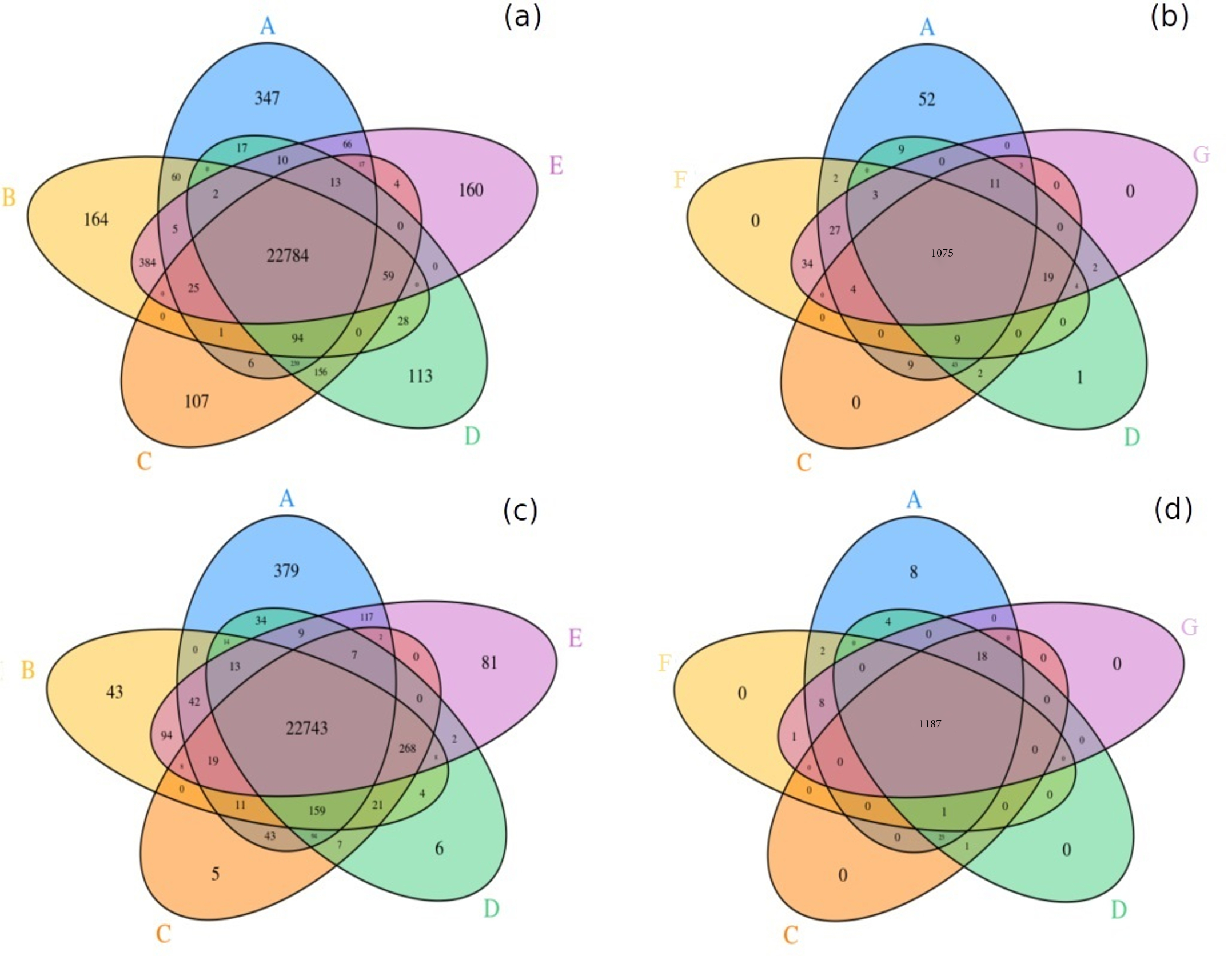

Supplement: Supplementary file 5 — Figure S5. Venn diagram depicting the comparison of top four pipelines. GiaB variants (A) compared against the top performing pipelines (B) BWA_SAMtools, (C) BWA_DeepVariant, (D) Novoalign_DeepVariant, (E) Novoalign_SAMtools, (F) BWA_GATK and (G) Novoalign_GATK for SNVs (a, c) and InDels (b, d) on exome-3 (top row) and exome-4 (bottom row). (PNG 2321 kb) [file 12859_2019_2928_MOESM5_ESM.png]
