# Supplementary material for: Performance assessment of variant calling pipelines using human whole exome sequencing and simulated data
Source: BMC Bioinformatics. 2019 Jun 17;20:342. doi: 10.1186/s12859-019-2928-9 (PMC6580603; doi:10.1186/s12859-019-2928-9)
Supplement: Supplementary file 6 — Table S1. Tools used in pipeline development. (PDF 217 kb) [file 12859_2019_2928_MOESM6_ESM.pdf]

Table S1. Tools used in pipeline development.

Tool	Type	Version	Algorithm	Features	Reference
**Burrows-Wheeler Aligner (BWA)**	Aligner	0.7.16	BWA-backtrack, BWA-SW and BWA-MEM* **Used in this study*	BWA-MEM and BWA-SW: long-read support and split alignment. BWA-MEM: faster and more accurate, and is for high-quality queries	H. Li, R. Durbin, Fast and accurate short read alignment with Burrows-Wheeler Transform. Bioinformatics, 25 (2009) 1754-1760
**Bowtie2**	Aligner	2.2.6	Burrows-Wheeler transform, FM-index	Finds longer, gapped alignments	B. Langmead, S.L. Salzberg, Fast gapped-read alignment with Bowtie 2, Nat. Methods. 9 (2012) 357-359.
**Novoalign**	Aligner	3.08.02	Hash table indexing/ Needleman-Wunsch algorithm	Slow, high memory usage, good for mapping with long gaps	http://www.novocraft.com
**SOAP**	Aligner	2.21	Burrows-Wheeler transformation	Speed and efficient memory usage	R. Li, Y. Li, K. Kristiansen, J. Wang, SOAP: short oligonucleotide alignment program, Bioinformatics, 24 (2008) 713-714.
**MOSAIK**	Aligner	2.2.3	K-mer hash index/ Smith-Waterman algorithm	Featured in a short-read, cross-species comparison, fast gaped alignment	W.P. Lee, M.P. Stromberg, A. Ward, C. Stewart, E.P. Garrison, G.T. Marth, MOSAIK: A Hash-Based Algorithm for Accurate Next-Generation Sequencing Short-Read Mapping. PLOS ONE 9 (2014) e90581.
**Genome Analysis Toolkit (GATK)**	Variant caller	4	Bayes' rule/ Smith-Waterman	Only applied for small InDel	https://software.broadinstitute.org/gatk/gatk4

**FreeBayes**	Variant caller	1.1.0	Bayes' rule	Separate caller for SNPs, indels, and multi-nucleotide polymorphisms. Utilized by the 1000 Genomes Project	E. Garrison, G. Marth, Haplotype-based variant detection from short-read sequencing, Cornell University Library, arXiv:1207.3907 [q-bio.GN], (2012).
**SAMtools mpileup**	Variant caller	1.7	MAQ model framework and general Bayesian framework	High positive prediction and simple use	H. Li, B. Handsaker, A. Wysoker, T. Fennell, J. Ruan, N. Homer, G. Marth, G. Abecasis, R. Durbin, 1000 Genome Project Data Processing Subgroup. The sequence alignment/map format and SAMtools, Bioinformatics. 25 (2009) 2078-2079.
**DeepVariant**	Variant caller	r0.4	Trained Convolutional Neural Network (CNN); stochastic gradient descent algorithm	statistical modeling towards more automatic deep learning approaches for developing software to interpret biological instrumentation data.	bioRxiv preprint first posted online Dec. 14, 2016; doi: http://dx.doi.org/10.1101/092890
