# Supplementary material for: Performance assessment of variant calling pipelines using human whole exome sequencing and simulated data
Source: BMC Bioinformatics. 2019 Jun 17;20:342. doi: 10.1186/s12859-019-2928-9 (PMC6580603; doi:10.1186/s12859-019-2928-9)
Supplement: Supplementary file 7 — Table S2. Run time (in min) of 20 variant calling pipelines. (PDF 186 kb) [file 12859_2019_2928_MOESM7_ESM.pdf]

Table S2. Run time (in min) of 20 variant calling pipeline

Pipeline	Exome-1	Exome-2	Exome-3	Exome-4	Multithreding-aligner (* available in commercial version)	Multithreding-Variant Caller
Bowtie_DeepVariant	2271	2314	2439	2436	Y	Y
Bowtie_FreeBayes	2163	2094	2039	2169	Y	N
Bowtie_GATK	2317	2489	2394	2393	Y	Y
Bowtie_SAMtools	2394	2394	2483	2511	Y	N
BWA_DeepVariant	2209	2397	2269	2393	Y	Y
BWA_FreeBayes	2008	1954	2193	2218	Y	N
BWA_GATK	2291	2089	2093	2189	Y	Y
BWA_SAMtools	2394	2463	2286	2176	Y	N
Mosaik_DeepVariant	2094	2375	2467	2199	Y	Y
MOSAIK_FreeBayes	2174	2197	2394	2376	Y	N
MOSAIK_GATK	2193	2294	2489	2547	Y	Y
MOSAIK_SAMtools	2395	2456	2463	2531	Y	N
Novoalign*_DeepVariant	3294	3521	3675	3812	Y*	Y
Novoalign*_FreeBayes	3217	3514	3591	3643	Y*	N
Novoalign*_GATK	3391	3581	3721	3843	Y*	Y
Novoalign*_SAMtools	3513	3617	3943	3899	Y*	N
SOAP_DeepVariant	2231	2392	2281	2563	Y	Y
SOAP_FreeBayes	2217	2346	2408	2317	Y	N
SOAP_GATK	2364	2324	2194	2493	Y	Y
SOAP_SAMtools	2509	2493	2376	2434	Y	N
