# Supplementary material for: Performance assessment of variant calling pipelines using human whole exome sequencing and simulated data
Source: BMC Bioinformatics. 2019 Jun 17;20:342. doi: 10.1186/s12859-019-2928-9 (PMC6580603; doi:10.1186/s12859-019-2928-9)
Supplement: Supplementary file 8 — Table S3. Performance of twenty pipelines. Performance of pipelines analyzed for SNVs and InDels on real (exome-1 and exome-3) and simulated exome data (exome-2 and exome-4). (PDF 386 kb) [file 12859_2019_2928_MOESM8_ESM.pdf]

Table S3. Performance of 20 pipelines.
Performance of pipelines analyzed for SNVs and InDels on real (exome-1 and exome-3) and simulated data (exome-2 and exome-4)

Exome_1	SNVs							InDels						
Pipeline	TP	FP	FN	Sensitivity	Precision	F-Score	FDR	TP	FP	FN	Sensitivity	Precision	F-Score	FDR
Bowtie_DeepVariant	23318	921	984	0.960	0.962	0.961	0.038	1122	109	207	0.844	0.911	0.877	0.082
Bowtie_FreeBayes	23426	2816	876	0.964	0.893	0.927	0.116	1086	142	243	0.817	0.884	0.849	0.107
Bowtie_GATK	23246	1562	1056	0.957	0.937	0.947	0.064	1159	51	170	0.872	0.958	0.913	0.038
Bowtie_SAMtools	23376	1008	926	0.962	0.959	0.960	0.041	1007	308	322	0.758	0.766	0.762	0.232
BWA_DeepVariant	23746	667	556	0.977	0.973	0.975	0.027	1252	47	77	0.942	0.964	0.953	0.035
BWA_FreeBayes	22812	1543	1490	0.939	0.937	0.938	0.063	1108	106	221	0.834	0.913	0.871	0.080
BWA_GATK	23689	1397	613	0.975	0.944	0.959	0.057	1254	72	75	0.944	0.946	0.945	0.054
BWA_SAMtools	23527	806	775	0.968	0.967	0.967	0.033	986	239	343	0.742	0.805	0.772	0.180
MOSAIK_DeepVariant	23300	873	1002	0.959	0.964	0.961	0.036	1153	94	176	0.868	0.925	0.895	0.071
MOSAIK_FreeBayes	22946	3157	1356	0.944	0.879	0.910	0.130	1097	395	232	0.825	0.735	0.778	0.297
MOSAIK_GATK	3194	463	21108	0.131	0.873	0.228	0.019	186	10	1143	0.140	0.949	0.244	0.008
MOSAIK_SAMtools	-	-	-	-	-	-	-		-	-	-	-	-	-
Novoalign_DeepVariant	23735	683	567	0.977	0.972	0.974	0.028	1255	53	74	0.944	0.959	0.952	0.040
Novoalign_FreeBayes	23156	3456	1146	0.953	0.870	0.910	0.142	1096	97	233	0.825	0.919	0.869	0.073
Novoalign_GATK	23637	1441	665	0.973	0.943	0.957	0.059	1255	67	74	0.944	0.949	0.947	0.050
Novoalign_SAMtools	23569	795	733	0.970	0.967	0.969	0.033	1019	196	310	0.767	0.839	0.801	0.147
SOAP_DeepVariant	-	-	-	-	-	-	-	-	-	-	-	-	-	-
SOAP_FreeBayes	22894	8064	1408	0.942	0.740	0.829	0.332	264	26	1065	0.199	0.910	0.326	0.020
SOAP_GATK	22186	416	2116	0.913	0.982	0.946	0.017	189	8	1140	0.142	0.959	0.248	0.006
SOAP_SAMtools	-	-	-	-	-	-	-	-	-	-	-	-	-	-
Exome_2	SNVs							InDels						
Bowtie_DeepVariant	23815	593	487	0.980	0.976	0.978	0.024	1190	10	139	0.895	0.992	0.941	0.008
Bowtie_FreeBayes	22866	1509	1436	0.941	0.938	0.939	0.062	1127	1286	202	0.848	0.467	0.602	0.968
Bowtie_GATK	22069	943	2233	0.908	0.959	0.933	0.039	1193	156	136	0.898	0.884	0.891	0.117
Bowtie_SAMtools	23754	638	548	0.977	0.974	0.976	0.026	1007	264	322	0.758	0.792	0.775	0.199
BWA_DeepVariant	24095	278	207	0.991	0.989	0.990	0.011	1316	2	13	0.990	0.998	0.994	0.002
BWA_FreeBayes	22945	487	1357	0.944	0.979	0.961	0.020	1139	1196	190	0.857	0.488	0.622	0.900
BWA_GATK	23946	865	356	0.985	0.965	0.975	0.036	1309	10	20	0.985	0.992	0.989	0.008
BWA_SAMtools	24008	487	294	0.988	0.980	0.984	0.020	997	294	332	0.750	0.772	0.761	0.221
MOSAIK_DeepVariant	23608	649	694	0.971	0.973	0.972	0.027	1131	26	198	0.851	0.978	0.910	0.020
MOSAIK_FreeBayes	21036	1489	3266	0.866	0.934	0.898	0.061	1093	1439	236	0.822	0.432	0.566	1.083
MOSAIK_GATK	21047	911	3255	0.866	0.959	0.910	0.037	1171	208	158	0.881	0.849	0.865	0.157
MOSAIK_SAMtools	-	-	-	-	-	-	-	-	-	-	-	-	-	-

**Novoalign_DeepVariant**	24107	264	195	0.992	0.989	0.991	0.011	1310	1	19	0.986	0.999	0.992	0.001
**Novoalign_FreeBayes**	21983	1375	2319	0.905	0.941	0.922	0.057	1156	1274	173	0.870	0.476	0.615	0.959
**Novoalign_GATK**	23967	813	335	0.986	0.967	0.977	0.033	1311	7	18	0.986	0.995	0.991	0.005
**Novoalign_SAMtools**	24011	397	291	0.988	0.984	0.986	0.016	966	264	363	0.727	0.785	0.755	0.199
**SOAP_DeepVariant**	-	-	-	-	-	-	-	-	-	-	-	-	-	-
**SOAP_FreeBayes**	21964	1375	2338	0.904	0.941	0.922	0.057	1099	1297	230	0.827	0.459	0.590	0.976
**SOAP_GATK**	23146	1069	1156	0.952	0.956	0.954	0.044	1178	293	151	0.886	0.801	0.841	0.220
**SOAP_SAMtools**	-	-	-	-	-	-	-	-	-	-	-	-	-	-
**Exome_3**	**SNVs**							**InDels**						
**Bowtie_DeepVariant**	22903	583	783	0.967	0.975	0.971	0.025	1126	93	132	0.895	0.924	0.909	0.074
**Bowtie_FreeBayes**	22831	2771	855	0.964	0.892	0.926	0.117	1036	154	222	0.824	0.871	0.846	0.122
**Bowtie_GATK**	22641	1436	1045	0.956	0.940	0.948	0.061	1102	45	156	0.876	0.961	0.916	0.036
**Bowtie_SAMtools**	22781	942	905	0.962	0.960	0.961	0.040	926	290	332	0.736	0.762	0.749	0.231
**BWA_DeepVariant**	23179	326	507	0.979	0.986	0.982	0.014	1165	21	93	0.926	0.982	0.953	0.017
**BWA_FreeBayes**	22812	1467	874	0.963	0.940	0.951	0.062	1026	90	232	0.816	0.919	0.864	0.072
**BWA_GATK**	23099	1523	587	0.975	0.938	0.956	0.064	1160	31	98	0.922	0.974	0.947	0.025
**BWA_SAMtools**	22971	635	715	0.970	0.973	0.971	0.027	966	223	292	0.768	0.812	0.790	0.177
**MOSAIK_DeepVariant**	22783	689	903	0.962	0.971	0.966	0.029	965	101	293	0.767	0.905	0.830	0.080
**MOSAIK_FreeBayes**	22715	3368	971	0.959	0.871	0.913	0.142	1032	355	226	0.820	0.744	0.780	0.282
**MOSAIK_GATK**	2056	347	21630	0.087	0.856	0.158	0.015	61	20	1197	0.048	0.753	0.091	0.016
**MOSAIK_SAMtools**	-	-	-	-	-	-	-	-	-	-	-	-	-	-
**Novoalign_DeepVariant**	23159	356	527	0.978	0.985	0.981	0.015	1161	28	97	0.923	0.976	0.949	0.022
**Novoalign_FreeBayes**	22688	3333	998	0.958	0.872	0.913	0.141	1029	84	229	0.818	0.925	0.868	0.067
**Novoalign_GATK**	23209	1591	477	0.980	0.936	0.957	0.067	1163	34	95	0.924	0.972	0.947	0.027
**Novoalign_SAMtools**	22922	407	764	0.968	0.983	0.975	0.017	971	179	287	0.772	0.844	0.806	0.142
**SOAP_DeepVariant**	-	-	-	-	-	-	-	-	-	-	-	-	-	-
**SOAP_FreeBayes**	22242	7862	1444	0.939	0.739	0.827	0.332	152	14	1106	0.121	0.916	0.213	0.011
**SOAP_GATK**	21943	221	1743	0.926	0.990	0.957	0.009	139	19	1119	0.110	0.880	0.196	0.015
**SOAP_SAMtools**	-	-	-	-	-	-	-	-	-	-	-	-	-	-
**Exome_4**	**SNVs**							**InDels**						
**Bowtie_DeepVariant**	22993	539	693	0.971	0.977	0.974	0.023	1102	32	156	0.876	0.972	0.921	0.025
**Bowtie_FreeBayes**	21110	1405	2576	0.891	0.938	0.914	0.059	1070	1063	188	0.851	0.502	0.631	0.845
**Bowtie_GATK**	21394	917	2292	0.903	0.959	0.930	0.039	1098	89	160	0.873	0.925	0.898	0.071
**Bowtie_SAMtools**	22840	553	846	0.964	0.976	0.970	0.023	878	189	380	0.698	0.823	0.755	0.150
**BWA_DeepVariant**	23078	309	608	0.974	0.987	0.981	0.013	1236	1	22	0.983	0.999	0.991	0.001
**BWA_FreeBayes**	22308	431	1378	0.942	0.981	0.961	0.018	1078	1047	180	0.857	0.507	0.637	0.832
**BWA_GATK**	23052	743	634	0.973	0.969	0.971	0.031	1234	2	24	0.981	0.998	0.990	0.002
**BWA_SAMtools**	23001	446	685	0.971	0.981	0.976	0.019	918	112	340	0.730	0.891	0.802	0.089
**MOSAIK_DeepVariant**	20702	645	2984	0.874	0.970	0.919	0.027	1051	64	207	0.835	0.943	0.886	0.051

**MOSAIK_FreeBayes**	20896	1340	2790	0.882	0.940	0.910	0.057	1067	1203	191	0.848	0.470	0.605	0.956
**MOSAIK_GATK**	20981	805	2705	0.886	0.963	0.923	0.034	1099	99	159	0.874	0.917	0.895	0.079
**MOSAIK_SAMtools**	-	-	-	-	-	-	-	-	-	-		-	-	-
**Novoalign_DeepVariant**	23073	316	613	0.974	0.986	0.980	0.013	1240	1	18	0.986	0.999	0.992	0.001
**Novoalign_FreeBayes**	21069	1253	2617	0.890	0.944	0.916	0.053	1090	1043	168	0.866	0.511	0.643	0.829
**Novoalign_GATK**	23037	843	649	0.973	0.965	0.969	0.036	1237	1	21	0.983	0.999	0.991	0.001
**Novoalign_SAMtools**	22952	377	734	0.969	0.984	0.976	0.016	924	178	334	0.734	0.838	0.783	0.141
**SOAP_DeepVariant**	-	-		-	-	-	-	-	-	-		-	-	-
**SOAP_FreeBayes**	21161	1256	2525	0.893	0.944	0.918	0.053	1054	1165	204	0.838	0.475	0.606	0.926
**SOAP_GATK**	22681	982	1005	0.958	0.959	0.958	0.041	1083	118	175	0.861	0.902	0.881	0.094
**SOAP_SAMtools**	-	-	-	-	-	-	-	-	-	-		-	-	-
