# Supplementary material for: Performance assessment of variant calling pipelines using human whole exome sequencing and simulated data
Source: BMC Bioinformatics. 2019 Jun 17;20:342. doi: 10.1186/s12859-019-2928-9 (PMC6580603; doi:10.1186/s12859-019-2928-9)
Supplement: Supplementary file 9 — Table S4. Performance (F-score) of pipelines with respect to genotype quality (GQ) for SNVs and InDels (PDF 383 kb) [file 12859_2019_2928_MOESM9_ESM.pdf]

Table S4. Performance (F-score) of pipelines with respect to genotype quality (GQ) for SNVs and InDels

Exome_1												
**GQ**	**0**	**20**	**40**	**60**	**80**	**100**	**0**	**20**	**40**	**60**	**80**	**100**
**Pipeline**	**SNVs**						**InDels**					
Bowtie_DeepVariant	0	0.29	0.53	0.63	0.71	0.96	0	0.24	0.39	0.51	0.57	0.88
Bowtie_FreeBayes	0	0.13	0.31	0.41	0.57	0.93	0	0.19	0.33	0.42	0.53	0.85
Bowtie_GATK	0	0.22	0.44	0.54	0.63	0.95	0	0.28	0.44	0.56	0.62	0.00
Bowtie_SAMtools	0	0.28	0.53	0.63	0.75	0.96	0	0.11	0.23	0.31	0.39	0.76
BWA_DeepVariant	0	0.41	0.65	0.73	0.8	0.97	0	0.46	0.64	0.72	0.77	0.95
BWA_FreeBayes	0	0.23	0.41	0.52	0.61	0.94	0	0.18	0.39	0.48	0.55	0.87
BWA_GATK	0	0.24	0.45	0.56	0.65	0.96	0	0.37	0.56	0.65	0.71	0.00
BWA_SAMtools	0	0.35	0.58	0.68	0.75	0.97	0	0.12	0.27	0.34	0.41	0.77
Mosaik_DeepVariant	0	0.29	0.53	0.64	0.71	0.96	0	0.25	0.45	0.54	0.63	0.90
MOSAIK_FreeBayes	0	0.15	0.32	0.42	0.51	0.91	0	0.11	0.23	0.32	0.38	0.78
MOSAIK_GATK	0	0.01	0.05	0.06	0.07	0.23	0	0.03	0.04	0.08	0.12	0.00
MOSAIK_SAMtools	-	-	-	-	-	-	-	-	-	-	-	-
Novoalign_DeepVariant	0	0.4	0.64	0.73	0.79	0.97	0	0.47	0.66	0.73	0.78	0.95
Novoalign_FreeBayes	0	0.14	0.32	0.42	0.52	0.91	0	0.21	0.38	0.48	0.55	0.87
Novoalign_GATK	0	0.23	0.44	0.55	0.63	0.96	0	0.37	0.57	0.66	0.71	0.00
Novoalign_SAMtools	0	0.35	0.59	0.69	0.76	0.97	0	0.14	0.29	0.37	0.44	0.80
SOAP_DeepVariant	-	-	-	-	-	-	-	-	-	-	-	-
SOAP_FreeBayes	0	0.07	0.18	0.26	0.33	0.83	0	0.04	0.05	0.09	0.15	0.33
SOAP_GATK	0	0.17	0.37	0.47	0.55	0.95	0	0.01	0.03	0.06	0.09	0.00
SOAP_SAMtools	-	-	-	-	-	-	-	-	-	-	-	-
**Exome_2**	**SNVs**						**InDels**					
Bowtie_DeepVariant	0	0.43	0.67	0.75	0.81	0.98	0	0.44	0.62	0.72	0.77	0.94
Bowtie_FreeBayes	0	0.21	0.42	0.53	0.61	0.94	0	0.06	0.12	0.19	0.24	0.60
Bowtie_GATK	0	0.19	0.39	0.53	0.59	0.93	0	0.24	0.39	0.51	0.61	0.00
Bowtie_SAMtools	0	0.41	0.65	0.74	0.8	0.98	0	0.14	0.25	0.39	0.45	0.77
BWA_DeepVariant	0	0.66	0.83	0.88	0.91	0.99	0	0.89	0.95	0.96	0.97	0.99
BWA_FreeBayes	0	0.28	0.54	0.64	0.72	0.96	0	0.06	0.14	0.19	0.26	0.62
BWA_GATK	0	0.29	0.52	0.62	0.72	0.00	0	0.66	0.81	0.86	0.89	0.00
BWA_SAMtools	0	0.54	0.75	0.82	0.87	0.98	0	0.16	0.28	0.37	0.45	0.76
Mosaik_DeepVariant	0	0.37	0.62	0.71	0.78	0.97	0	0.31	0.52	0.62	0.65	0.91
MOSAIK_FreeBayes	0	0.14	0.31	0.41	0.49	0.90	0	0.05	0.11	0.17	0.19	0.57

MOSAIK_GATK	0	0.15	0.33	0.43	0.52	0.00	0	0.19	0.36	0.47	0.51	0.00
MOSAIK_SAMtools	-	-	-	-	-	-	-	-	-	-	-	-
Novoalign_DeepVariant	0	0.67	0.84	0.89	0.92	0.99	0	0.87	0.93	0.95	0.96	0.99
Novoalign_FreeBayes	0	0.17	0.37	0.47	0.56	0.92	0	0.05	0.13	0.19	0.24	0.62
Novoalign_GATK	0	0.29	0.53	0.63	0.71	0.00	0	0.65	0.81	0.85	0.89	0.00
Novoalign_SAMtools	0	0.54	0.75	0.82	0.87	0.99	0	0.16	0.29	0.37	0.45	0.75
SOAP_DeepVariant	-	-	-	-	-	-	-	-	-	-	-	-
SOAP_FreeBayes	0	0.17	0.37	0.47	0.56	0.92	0	0.06	0.12	0.19	0.23	0.59
SOAP_GATK	0	0.24	0.47	0.58	0.66	0.00	0	0.18	0.32	0.43	0.48	0.00
SOAP_SAMtools	-	-	-	-	-	-	-	-	-	-	-	-
**Exome_3**	**SNVs**						**InDels**					
Bowtie_DeepVariant	0	0.37	0.61	0.71	0.77	0.97	0	0.22	0.38	0.49	0.55	0.91
Bowtie_FreeBayes	0	0.16	0.37	0.47	0.57	0.93	0	0.14	0.31	0.42	0.51	0.85
Bowtie_GATK	0	0.22	0.44	0.55	0.63	0.00	0	0.24	0.36	0.54	0.62	0.00
Bowtie_SAMtools	0	0.29	0.54	0.64	0.71	0.96	0	0.09	0.22	0.31	0.36	0.75
BWA_DeepVariant	0	0.51	0.73	0.79	0.85	0.98	0	0.41	0.64	0.71	0.76	0.95
BWA_FreeBayes	0	0.25	0.48	0.58	0.66	0.95	0	0.16	0.36	0.47	0.53	0.86
BWA_GATK	0	0.24	0.46	0.57	0.65	0.96	0	0.32	0.55	0.64	0.69	0.00
BWA_SAMtools	0	0.38	0.62	0.71	0.78	0.97	0	0.13	0.23	0.37	0.43	0.79
Mosaik_DeepVariant	0	0.32	0.58	0.67	0.74	0.97	0	0.11	0.28	0.47	0.63	0.83
MOSAIK_FreeBayes	0	0.15	0.33	0.43	0.52	0.91	0	0.09	0.22	0.31	0.36	0.78
MOSAIK_GATK	0	0.01	0.05	0.06	0.07	0.00	0	0.02	0.04	0.06	0.08	0.00
MOSAIK_SAMtools	-	-	-	-	-	-	-	-	-	-	-	-
Novoalign_DeepVariant	0	0.48	0.71	0.79	0.84	0.98	0	0.16	0.32	0.39	0.45	0.95
Novoalign_FreeBayes	0	0.15	0.33	0.43	0.52	0.91	0	0.17	0.38	0.48	0.55	0.87
Novoalign_GATK	0	0.22	0.44	0.55	0.63	0.96	0	0.34	0.55	0.65	0.78	0.00
Novoalign_SAMtools	0	0.37	0.61	0.71	0.77	0.98	0	0.41	0.62	0.72	0.75	0.81
SOAP_DeepVariant	-	-	-	-	-	-	-	-	-	-	-	-
SOAP_FreeBayes	0	0.07	0.18	0.26	0.33	0.83	0	0.03	0.04	0.09	0.12	0.21
SOAP_GATK	0	0.19	0.44	0.51	0.59	0.00	0	0.01	0.02	0.03	0.04	0.00
SOAP_SAMtools	-	-	-	-	-	-	-	-	-	-	-	-
**Exome_4**	**SNVs**						**InDels**					
Bowtie_DeepVariant	0	0.39	0.64	0.73	0.79	0.97	0	0.32	0.54	0.63	0.7	0.92
Bowtie_FreeBayes	0	0.15	0.35	0.45	0.54	0.91	0	0.05	0.13	0.19	0.24	0.63
Bowtie_GATK	0	0.19	0.38	0.49	0.58	0.00	0	0.21	0.39	0.5	0.58	0.00
Bowtie_SAMtools	0	0.36	0.61	0.72	0.77	0.97	0	0.12	0.24	0.35	0.43	0.76
BWA_DeepVariant	0	0.48	0.71	0.79	0.84	0.98	0	0.82	0.91	0.94	0.95	0.99
BWA_FreeBayes	0	0.28	0.54	0.65	0.72	0.96	0	0.06	0.13	0.2	0.26	0.64

BWA_GATK	0	0.27	0.51	0.61	0.69	0.97	0	0.65	0.81	0.87	0.9	0.00
BWA_SAMtools	0	0.42	0.66	0.75	0.81	0.98	0	0.17	0.36	0.42	0.52	0.80
Mosaik_DeepVariant	0	0.18	0.38	0.48	0.56	0.92	0	0.23	0.42	0.53	0.58	0.89
MOSAIK_FreeBayes	0	0.16	0.34	0.44	0.53	0.91	0	0.05	0.11	0.17	0.22	0.60
MOSAIK_GATK	0	0.15	0.33	0.43	0.51	0.00	0	0.22	0.42	0.51	0.57	0.00
MOSAIK_SAMtools	-	-	-	-	-	-	-	-	-	-	-	-
Novoalign_DeepVariant	0	0.48	0.71	0.78	0.84	0.98	0	0.83	0.93	0.95	0.96	0.99
Novoalign_FreeBayes	0	0.17	0.36	0.46	0.55	0.92	0	0.05	0.15	0.22	0.27	0.64
Novoalign_GATK	0	0.26	0.49	0.61	0.67	0.97	0	0.72	0.85	0.9	0.92	0.00
Novoalign_SAMtools	0	0.41	0.65	0.74	0.82	0.98	0	0.17	0.27	0.39	0.47	0.78
SOAP_DeepVariant	-	-	-	-	-		-	-	-	-	-	-
SOAP_FreeBayes	0	0.17	0.36	0.47	0.55	0.92	0	0.05	0.12	0.22	0.33	0.61
SOAP_GATK	0	0.25	0.49	0.59	0.67	0.00	0	0.22	0.44	0.54	0.66	0.00
SOAP_SAMtools	-	-	-	-	-	-	-	-	-	-	-	-
