# Supplementary material for: Performance assessment of variant calling pipelines using human whole exome sequencing and simulated data
Source: BMC Bioinformatics. 2019 Jun 17;20:342. doi: 10.1186/s12859-019-2928-9 (PMC6580603; doi:10.1186/s12859-019-2928-9)
Supplement: Supplementary file 10 — Table S5. F-score of pipelines for SNVs and InDels. F-score was used as the function of genotype concordance (PDF 139 kb) [file 12859_2019_2928_MOESM10_ESM.pdf]

Table S5. F-score of pipelines for SNVs and InDels. F-score was used as the function of genotype concordance

	InDels detection				SNVs detection			
**Pipeline**	**Exome-1**	**Exome-3**	**Exome-4**	**Exome-4**	**Exome-1**	**Exome-2**	**Exome-3**	**Exome-4**
Bowtie_DeepVariant	0.884	0.908	0.914	0.914	0.873	0.893	0.893	0.913
Bowtie_FreeBayes	0.813	0.841	0.864	0.864	0.793	0.8186	0.805	0.834
Bowtie_GATK	0.845	0.893	0.905	0.905	0.816	0.829	0.843	0.893
Bowtie_SAMtools	0.756	0.767	0.816	0.816	0.856	0.863	0.864	0.893
BWA_DeepVariant	0.915	0.934	0.961	0.961	0.917	0.947	0.923	0.953
BWA_FreeBayes	0.806	0.849	0.864	0.864	0.843	0.863	0.843	0.861
BWA_GATK	0.893	0.92	0.946	0.946	0.853	0.883	0.883	0.891
BWA_SAMtools	0.743	0.756	0.805	0.805	0.903	0.934	0.913	0.943
Mosaik_DeepVariant	0.884	0.905	0.913	0.913	0.821	0.872	0.858	0.876
MOSAIK_FreeBayes	0.817	0.847	0.864	0.864	0.761	0.813	0.803	0.856
MOSAIK_GATK	0.143	0.158	0.834	0.834	0.784	0.838	0.823	0.851
MOSAIK_SAMtools	-	-	-	-	-	-	-	-
Novoalign_DeepVariant	0.911	0.929	0.958	0.958	0.921	0.951	0.919	0.949
Novoalign_FreeBayes	0.824	0.851	0.874	0.874	0.884	0.893	0.865	0.892
Novoalign_GATK	0.901	0.92	0.941	0.941	0.899	0.907	0.873	0.901
Novoalign_SAMtools	0.743	0.752	0.801	0.801	0.899	0.929	0.909	0.938
SOAP_DeepVariant	-	-	-	-	-	-	-	-
SOAP_FreeBayes	0.138	0.194	0.816	0.816	0.873	0.892	0.863	0.893
SOAP_GATK	0.056	0.062	0.864	0.864	0.846	0.861	0.823	0.843
SOAP_SAMtools	-	-	-	-	-	-	-	-
