# Supplementary material for: Performance assessment of variant calling pipelines using human whole exome sequencing and simulated data
Source: BMC Bioinformatics. 2019 Jun 17;20:342. doi: 10.1186/s12859-019-2928-9 (PMC6580603; doi:10.1186/s12859-019-2928-9)
Supplement: Supplementary file 11 — Table S6. F-score of pipelines for heterozygous and homozygous variants, and Heterozygous/Homozygous ratio. (PDF 290 kb) [file 12859_2019_2928_MOESM11_ESM.pdf]

Table S6. F-score of pipelines for heterozygous and homozygous variants, and Heterozygous/Homozygous

**SNVs**												
**Pipeline**	**Heterozygous (F-score)**				**Homozygous (F-score)**				**Het/Hom Ratio**			
	**Exome_1**	**Exome_2**	**Exome_3**	**Exome_4**	**Exome_1**	**Exome_2**	**Exome_3**	**Exome_4**	**Exome_1**	**Exome_2**	**Exome_3**	**Exome_4**
Bowtie_DeepVariant	0.96	0.98	0.96	0.97	0.97	0.97	0.98	0.98	1.59	1.61	1.49	1.49
Bowtie_FreeBayes	0.90	0.95	0.92	0.91	0.91	0.94	0.94	0.92	1.59	1.61	1.49	1.49
Bowtie_GATK	0.94	0.92	0.94	0.92	0.95	0.93	0.95	0.94	1.59	1.61	1.49	1.49
Bowtie_SAMtools	0.96	0.98	0.95	0.96	0.97	0.97	0.97	0.98	1.59	1.61	1.49	1.49
BWA_DeepVariant	0.97	0.99	0.98	0.97	0.98	0.99	0.99	0.99	1.59	1.61	1.49	1.49
BWA_FreeBayes	0.93	0.95	0.94	0.95	0.95	0.96	0.96	0.97	1.59	1.61	1.49	1.49
BWA_GATK	0.96	0.98	0.96	0.97	0.97	0.98	0.99	0.98	1.59	1.61	1.49	1.49
BWA_SAMtools	0.96	0.99	0.97	0.97	0.98	0.98	0.98	0.99	1.59	1.61	1.49	1.49
Mosaik_DeepVariant	0.96	0.98	-	-	0.97	0.97	-	-	1.59	1.61	-	-
MOSAIK_FreeBayes	0.91	0.89	0.91	0.90	0.92	0.89	0.92	0.92	1.59	1.61	1.49	1.49
MOSAIK_GATK	0.15	0.89	0.13	0.90	0.16	0.90	0.13	0.91	1.59	1.61	1.49	1.49
MOSAIK_SAMtools	-	-	0.96	0.91	-	-	0.98	0.93	-	-	1.49	1.49
Novoalign_DeepVariant	0.97	0.99	0.96	0.97	0.98	0.98	0.98	0.98	1.59	1.61	1.49	1.49
Novoalign_FreeBayes	0.91	0.92	0.91	0.91	0.92	0.92	0.92	0.93	1.59	1.61	1.49	1.49
Novoalign_GATK	0.96	0.98	0.95	0.97	0.97	0.96	0.98	0.97	1.59	1.61	1.49	1.49
Novoalign_SAMtools	0.96	0.99	0.97	0.97	0.98	0.99	0.99	0.99	1.59	1.61	1.49	1.49
SOAP_DeepVariant	-	-	-	-	-	-	-	-	-	-	-	-
SOAP_FreeBayes	0.82	0.92	0.82	0.91	0.83	0.92	0.84	0.93	1.59	1.61	1.49	1.49
SOAP_GATK	0.94	0.96	0.95	0.94	0.95	0.95	0.96	0.96	1.59	1.61	1.49	1.49
SOAP_SAMtools	-	-	-	-	-	-	-	-	-	-	-	-
**InDels**												
**Pipeline**	**Heterozygous (F-score)**				**Homozygous (F-score)**				**Het/Hom Ratio**			
	**Exome_1**	**Exome_2**	**Exome_3**	**Exome_4**	**Exome_1**	**Exome_2**	**Exome_3**	**Exome_4**	**Exome_1**	**Exome_2**	**Exome_3**	**Exome_4**
Bowtie_DeepVariant	0.87	0.94	0.88	0.92	0.88	0.94	0.94	0.93	1.19	1.19	1.16	1.26
Bowtie_FreeBayes	0.84	0.56	0.82	0.60	0.86	0.66	0.88	0.68	1.19	1.19	1.16	1.26
Bowtie_GATK	0.90	0.86	0.89	0.86	0.90	0.89	0.94	0.88	1.19	1.19	1.16	1.25
Bowtie_SAMtools	0.75	0.76	0.72	0.74	0.78	0.79	0.78	0.77	1.19	1.19	1.16	1.26
BWA_DeepVariant	0.95	1.00	0.93	0.99	0.96	0.99	0.98	1.00	1.19	1.19	1.16	1.26
BWA_FreeBayes	0.87	0.58	0.84	0.60	0.88	0.67	0.89	0.68	1.19	1.19	1.16	1.26
BWA_GATK	0.94	0.98	0.90	0.97	0.95	0.98	0.97	0.99	1.19	1.19	1.16	1.26
BWA_SAMtools	0.76	0.75	0.76	0.79	0.79	0.78	0.82	0.82	1.19	1.19	1.16	1.26
Mosaik_DeepVariant	0.89	0.91	-	-	0.90	0.91	-	-	1.19	1.19	-	-
MOSAIK_FreeBayes	0.76	0.53	0.75	0.57	0.80	0.62	0.82	0.65	1.19	1.19	1.16	1.26
MOSAIK_GATK	0.15	0.84	0.09	0.88	0.15	0.87	0.10	0.90	1.18	1.19	1.18	1.26
MOSAIK_SAMtools	-	-	0.81	0.88	-	-	0.86	0.90	-	-	1.16	1.26
Novoalign_DeepVariant	0.95	0.99	0.93	0.99	0.96	0.99	0.98	1.00	1.19	1.19	1.16	1.25
Novoalign_FreeBayes	0.86	0.58	0.84	0.61	0.88	0.67	0.90	0.69	1.19	1.19	1.16	1.26
Novoalign_GATK	0.94	0.99	0.92	0.98	0.96	0.99	0.98	0.99	1.19	1.19	1.16	1.26
Novoalign_SAMtools	0.78	0.74	0.78	0.77	0.92	0.77	0.84	0.80	1.19	1.19	1.16	1.26
SOAP_DeepVariant	-	-	-	-	-	-	-	-	-	-	-	-
SOAP_FreeBayes	0.33	0.55	0.21	0.57	0.33	0.64	0.22	0.65	1.18	1.19	1.17	1.26
SOAP_GATK	0.10	0.82	0.06	0.89	0.10	0.85	0.06	0.91	1.22	1.19	1.17	1.26
SOAP_SAMtools	-	-	-	-	-	-	-	-	-	-	-	-
