# Supplementary material for: Performance assessment of variant calling pipelines using human whole exome sequencing and simulated data
Source: BMC Bioinformatics. 2019 Jun 17;20:342. doi: 10.1186/s12859-019-2928-9 (PMC6580603; doi:10.1186/s12859-019-2928-9)
Supplement: Supplementary file 12 — Table S7. F-score of pipelines for transition and transversion detection, and Ti/Tv ratio. (PDF 215 kb) [file 12859_2019_2928_MOESM12_ESM.pdf]

Table S7. Performance (F-score) pipelines in transition and transversion detection, and Ti/Tv ratio

**Exome_1**					
**Pipe line**	**Transition count**	**Transition F-score**	**Transversion count**	**Transversion F-score**	**Ti/Tv ratio**
Bowtie_DeepVariant	18016	0.96	5302	0.96	3.40
Bowtie_FreeBayes	17327	0.93	5099	0.96	3.40
Bowtie_GATK	1792	0.94	5274	0.95	3.40
Bowtie_SAMTools	18061	0.96	5315	0.96	3.40
BWA_DeepVariant	18347	0.97	5399	0.98	3.40
BWA_FreeBayes	17625	0.94	5187	0.94	3.40
BWA_GATK	18292	0.95	5397	0.96	3.40
BWA_SAMTools	18177	0.97	5350	0.97	3.40
Mosaik_DeepVariant	18002	0.96	5298	0.96	3.40
Mosaik_FreeBayes	17729	0.91	5217	0.94	3.40
Mosaik_GATK	1724	0.15	1470	0.08	3.39
Mosaik_SAMTools	-	-	-	-	-
Novoalign_DeepVariant	18338	0.97	5397	0.98	3.40
Novoalign_FreeBayes	17891	0.91	5265	0.95	3.40
Novoalign_GATK	18236	0.94	5401	0.96	3.40
Novoalign_SAMTools	18210	0.97	5359	0.97	3.40
SOAP_DeepVariant	-	-	-	-	-
SOAP_FreeBayes	17688	0.83	5206	0.94	3.40
SOAP_GATK	17170	0.94	5016	0.91	3.40
SOAP_SAMTools	-	-	-	-	-
**Exome_2**					
**Pipe line**	**Transition count**	**Transition F_score**	**Transversion count**	**Transversion F_score**	**Ti/Tv ratio**
Bowtie_DeepVariant	18400	0.98	5415	0.98	3.40
Bowtie_FreeBayes	17667	0.94	5199	0.94	3.40

Bowtie_GATK	16979	0.93	4997	0.96	3.40
Bowtie_SAMTools	18446	0.98	5401	0.97	3.40
BWA_DeepVariant	18616	0.99	5479	0.99	3.40
BWA_FreeBayes	17728	0.96	5217	0.98	3.40
BWA_GATK	18487	0.96	5459	0.94	3.40
BWA_SAMTools	18549	0.98	5459	0.98	3.40
Mosaik_DeepVariant	18240	0.97	5368	0.97	3.40
Mosaik_FreeBayes	16253	0.9	4783	0.93	3.40
Mosaik_GATK	16282	0.95	4765	0.96	3.40
Mosaik_SAMTools	-	-	-	-	-
Novoalign_DeepVariant	18626	0.99	5481	0.98	3.40
Novoalign_FreeBayes	16984	0.94	4999	0.94	3.40
Novoalign_GATK	18504	0.96	5463	0.94	3.40
Novoalign_SAMTools	18551	0.99	5460	0.99	3.40
SOAP_DeepVariant	-	-	-	-	-
SOAP_FreeBayes	16970	0.95	4994	0.94	3.40
SOAP_GATK	17914	0.95	5232	0.95	3.40
SOAP_SAMTools	-	-	-	-	-
**Exome_3**					
**Pipe line**	**Transition count**	**Transition F_score**	**Transversion count**	**Transversion F_score**	**Ti/Tv ratio**
Bowtie_DeepVariant	17492	0.96	5411	0.97	3.23
Bowtie_FreeBayes	17479	0.93	5352	0.93	3.27
Bowtie_GATK	17416	0.94	5225	0.95	3.32
Bowtie_SAMTools	17437	0.96	5344	0.97	3.26
BWA_DeepVariant	17634	0.97	5545	0.98	3.18
BWA_FreeBayes	17467	0.95	5345	0.96	3.27
BWA_GATK	17640	0.94	5459	0.95	3.24
BWA_SAMTools	17512	0.97	5459	0.98	3.21
Mosaik_DeepVariant	17446	0.95	5337	0.96	3.27
Mosaik_FreeBayes	17362	0.91	5353	0.93	3.24

Mosaik_GATK	1544	0.23	512	0.12	2.23
Mosaik_SAMTools	-	-	-	-	-
Novoalign_DeepVariant	17576	0.97	5583	0.98	3.15
Novoalign_FreeBayes	17376	0.91	5312	0.91	3.27
Novoalign_GATK	17642	0.95	5567	0.92	3.27
Novoalign_SAMTools	17504	0.96	5418	0.97	3.23
SOAP_DeepVariant	-	-	-	-	-
SOAP_FreeBayes	17094	0.82	5148	0.84	3.32
SOAP_GATK	17176	0.94	4767	0.89	3.55
SOAP_SAMTools	-	-	-	-	-
**Exome_4**					
**Pipe line**	**Transition count**	**Transition F_score**	**Transversion count**	**Transversion F_score**	**Ti/Tv ratio**
Bowtie_DeepVariant	17446	0.94	5457	0.98	3.20
Bowtie_FreeBayes	16163	0.89	4947	0.95	3.27
Bowtie_GATK	16355	0.91	5039	0.96	3.19
Bowtie_SAMTools	17407	0.94	5433	0.98	3.20
BWA_DeepVariant	17647	0.97	5532	0.99	3.19
BWA_FreeBayes	17287	0.89	5198	0.98	3.29
BWA_GATK	17364	0.91	5511	0.97	3.21
BWA_SAMTools	17479	0.96	5522	0.99	3.17
Mosaik_DeepVariant	17394	0.93	5389	0.97	3.23
Mosaik_FreeBayes	15929	0.88	4967	0.96	3.21
Mosaik_GATK	16898	0.93	4083	0.97	3.94
Mosaik_SAMTools	-	-	-	-	-
Novoalign_DeepVariant	17597	0.97	5562	0.99	3.16
Novoalign_FreeBayes	16153	0.88	4916	0.96	3.29
Novoalign_GATK	17557	0.93	5480	0.97	3.23
Novoalign_SAMTools	17463	0.96	5459	0.99	3.20
SOAP_DeepVariant	-	-	-	-	-
SOAP_FreeBayes	16134	0.87	5027	0.97	3.21

SOAP_GATK	17506	0.89	5175	0.97	3.33
SOAP_SAMTools	-	-	-	-	-
