# Supplementary material for: Performance assessment of variant calling pipelines using human whole exome sequencing and simulated data
Source: BMC Bioinformatics. 2019 Jun 17;20:342. doi: 10.1186/s12859-019-2928-9 (PMC6580603; doi:10.1186/s12859-019-2928-9)
Supplement: Supplementary file 14 — Table S9. Performance of 20 pipelines on NA24385 and NA24631 data sets using both reference genomes GRCh38 and GRCh37 for SNVs and InDels. (PDF 420 kb) [file 12859_2019_2928_MOESM14_ESM.pdf]

Table S9. Performance of 20 pipelines on NA24385 and NA24631 data sets using both reference genomes GRCh38 and GRCh37 for SNVs and InDels

GRCh37														
NA24385														
	SNV							InDel						
**Pipeline**	**TP**	**FP**	**FN**	**Sensitivity**	**Precision**	**F-Score**	**FDR**	**TP**	**FP**	**FN**	**Sensitivity**	**Precision**	**F-Score**	**FDR**
**Bowtie_DeepVariant**	39912	663	1005	0.975	0.984	0.980	0.016	3066	254	188	0.942	0.923	0.933	0.077
**Bowtie_FreeBayes**	40001	574	970	0.976	0.986	0.981	0.014	2791	529	261	0.914	0.841	0.876	0.159
**Bowtie_GATK**	39901	674	986	0.976	0.983	0.980	0.017	3089	231	168	0.948	0.930	0.939	0.070
**Bowtie_SAMtools**	39967	608	993	0.976	0.985	0.980	0.015	2591	729	308	0.894	0.780	0.833	0.220
**BWA_DeepVariant**	40105	470	857	0.979	0.988	0.984	0.012	3145	175	121	0.963	0.947	0.955	0.053
**BWA_FreeBayes**	39537	1038	1837	0.956	0.974	0.965	0.026	3013	307	182	0.943	0.908	0.925	0.092
**BWA_GATK**	40089	486	897	0.978	0.988	0.983	0.012	3157	163	127	0.961	0.951	0.956	0.049
**BWA_SAMtools**	40091	484	911	0.978	0.988	0.983	0.012	2607	713	362	0.878	0.785	0.829	0.215
**MOSAIK_DeepVariant**	39909	666	1002	0.976	0.984	0.980	0.016	3061	259	179	0.945	0.922	0.933	0.078
**MOSAIK_FreeBayes**	39885	690	1871	0.955	0.983	0.969	0.017	2997	323	190	0.940	0.903	0.921	0.097
**MOSAIK_GATK**	7979	32596	158	0.981	0.197	0.328	0.803	1501	1819	1820	0.452	0.452	0.452	0.548
**MOSAIK_SAMtools**	-	-	-	-	-	-	-	-	-	-	-	-	-	-
**Novoalign_DeepVariant**	40098	477	863	0.979	0.988	0.984	0.012	3189	131	103	0.969	0.961	0.965	0.039
**Novoalign_FreeBayes**	39881	694	1046	0.974	0.983	0.979	0.017	2984	336	205	0.936	0.899	0.917	0.101
**Novoalign_GATK**	40071	504	904	0.978	0.988	0.983	0.012	3183	137	111	0.966	0.959	0.963	0.041
**Novoalign_SAMtools**	40093	482	893	0.978	0.988	0.983	0.012	2655	665	279	0.905	0.800	0.849	0.200
**SOAP_DeepVariant**	-	-	-	-	-	-	-	-	-	-	-	-	-	-
**SOAP_FreeBayes**	39742	833	1806	0.957	0.979	0.968	0.021	1530	1790	1539	0.499	0.461	0.479	0.539
**SOAP_GATK**	39511	1064	1901	0.954	0.974	0.964	0.026	1518	1802	1639	0.481	0.457	0.469	0.543
**SOAP_SAMtools**	-	-	-	-	-	-	-	-	-	-	-	-	-	-

GRCh38														
NA24385														
	SNV							InDel						
**Pipeline**	**TP**	**FP**	**FN**	**Sensitivity**	**Precision**	**F-Score**	**FDR**	**TP**	**FP**	**FN**	**Sensitivity**	**Precision**	**F-Score**	**FDR**
**Bowtie_DeepVariant**	41071	1769	1099	0.974	0.959	0.966	0.041	4028	484	365	0.917	0.893	0.905	0.107
**Bowtie_FreeBayes**	40921	1919	1231	0.971	0.955	0.963	0.045	3955	557	401	0.908	0.877	0.892	0.123
**Bowtie_GATK**	41090	1750	1148	0.973	0.959	0.966	0.041	4021	491	312	0.928	0.891	0.909	0.109
**Bowtie_SAMtools**	42103	737	1071	0.975	0.983	0.979	0.017	3882	630	476	0.891	0.860	0.875	0.140
**BWA_DeepVariant**	42267	573	1031	0.976	0.987	0.981	0.013	4101	411	261	0.940	0.909	0.924	0.091
**BWA_FreeBayes**	42063	777	1261	0.971	0.982	0.976	0.018	4001	511	371	0.915	0.887	0.901	0.113
**BWA_GATK**	42152	688	1081	0.975	0.984	0.979	0.016	4095	417	278	0.936	0.908	0.922	0.092
**BWA_SAMtools**	42257	583	1049	0.976	0.986	0.981	0.014	3861	651	498	0.886	0.856	0.870	0.144
**MOSAIK_DeepVariant**	41082	1758	1111	0.974	0.959	0.966	0.041	4011	501	325	0.925	0.889	0.907	0.111
**MOSAIK_FreeBayes**	42077	763	1293	0.970	0.982	0.976	0.018	3967	545	355	0.918	0.879	0.898	0.121
**MOSAIK_GATK**	13830	29010	941	0.936	0.323	0.480	0.677	3561	951	2094	0.630	0.789	0.701	0.211
**MOSAIK_SAMtools**	-	-	-	-	-	-	-	-	-	-	-	-	-	-
**Novoalign_DeepVariant**	42274	566	1023	0.976	0.987	0.982	0.013	4131	381	234	0.946	0.916	0.931	0.084
**Novoalign_FreeBayes**	42051	789	1290	0.970	0.982	0.976	0.018	3951	561	392	0.910	0.876	0.892	0.124
**Novoalign_GATK**	42139	701	1079	0.975	0.984	0.979	0.016	4129	383	241	0.945	0.915	0.930	0.085
**Novoalign_SAMtools**	42251	589	1059	0.976	0.986	0.981	0.014	3897	615	420	0.903	0.864	0.883	0.136
**SOAP_DeepVariant**	-	-	-	-	-	-	-	-	-	-	-	-	-	-
**SOAP_FreeBayes**	40123	2717	2124	0.950	0.937	0.943	0.063	3592	920	2042	0.638	0.796	0.708	0.204
**SOAP_GATK**	40891	1949	2041	0.952	0.955	0.953	0.045	3402	1110	2104	0.618	0.754	0.679	0.246
**SOAP_SAMtools**	-	-	-	-	-	-	-	-	-	-	-	-	-	-
GRCh37														
NA24631														
	SNV							InDel						
**Pipeline**	**TP**	**FP**	**FN**	**Sensitivity**	**Precision**	**F-Score**	**FDR**	**TP**	**FP**	**FN**	**Sensitivity**	**Precision**	**F-Score**	**FDR**
**Bowtie_DeepVariant**	40482	649	992	0.976	0.984	0.980	0.016	2783	207	197	0.934	0.931	0.932	0.069
**Bowtie_FreeBayes**	40412	719	882	0.979	0.983	0.981	0.017	2421	569	280	0.896	0.810	0.851	0.190
**Bowtie_GATK**	40241	890	1063	0.974	0.978	0.976	0.022	2542	448	214	0.922	0.850	0.885	0.150

**Bowtie_SAMtools**	40462	669	1003	0.976	0.984	0.980	0.016	2391	599	318	0.883	0.800	0.839	0.200
**BWA_DeepVariant**	40580	551	748	0.982	0.987	0.984	0.013	2883	107	142	0.953	0.964	0.959	0.036
**BWA_FreeBayes**	40089	1042	2833	0.934	0.975	0.954	0.025	2481	509	259	0.905	0.830	0.866	0.170
**BWA_GATK**	40543	588	781	0.981	0.986	0.983	0.014	2858	132	181	0.940	0.956	0.948	0.044
**BWA_SAMtools**	40559	572	751	0.982	0.986	0.984	0.014	2309	681	1078	0.682	0.772	0.724	0.228
**MOSAIK_DeepVariant**	40213	918	1062	0.974	0.978	0.976	0.022	2691	299	203	0.930	0.900	0.915	0.100
**MOSAIK_FreeBayes**	40132	999	2041	0.952	0.976	0.964	0.024	2313	677	294	0.887	0.774	0.827	0.226
**MOSAIK_GATK**	8320	32811	430	0.951	0.202	0.334	0.798	2013	977	924	0.685	0.673	0.679	0.327
**MOSAIK_SAMtools**	-	-	-	-	-	-	-	-	-	-	-	-	-	-
**Novoalign_DeepVariant**	40583	548	734	0.982	0.987	0.984	0.013	2881	109	159	0.948	0.964	0.956	0.036
**Novoalign_FreeBayes**	40092	1039	2174	0.949	0.975	0.961	0.025	2311	679	274	0.894	0.773	0.829	0.227
**Novoalign_GATK**	40537	594	821	0.980	0.986	0.983	0.014	2866	124	179	0.941	0.959	0.950	0.041
**Novoalign_SAMtools**	40563	568	739	0.982	0.986	0.984	0.014	2351	639	351	0.870	0.786	0.826	0.214
**SOAP_DeepVariant**	-	-	-	-	-	-	-	-	-	-	-	-	-	-
**SOAP_FreeBayes**	40051	1080	2641	0.938	0.974	0.956	0.026	2308	682	1031	0.691	0.772	0.729	0.228
**SOAP_GATK**	40031	1100	3021	0.930	0.973	0.951	0.027	2129	861	953	0.691	0.712	0.701	0.288
**SOAP_SAMtools**	-	-	-	-	-	-	-	-	-	-	-	-	-	-
**GRCh38**														
**NA24631**														
	**SNV**							**InDel**						
**Pipeline**	**TP**	**FP**	**FN**	**Sensitivity**	**Precision**	**F-Score**	**FDR**	**TP**	**FP**	**FN**	**Sensitivity**	**Precision**	**F-Score**	**FDR**
**Bowtie_DeepVariant**	43492	837	1850	0.959	0.981	0.970	0.019	3412	227	562	0.859	0.938	0.896	0.062
**Bowtie_FreeBayes**	43351	978	1946	0.957	0.978	0.967	0.022	3333	306	653	0.836	0.916	0.874	0.084
**Bowtie_GATK**	43539	790	1831	0.960	0.982	0.971	0.018	3409	230	582	0.854	0.937	0.894	0.063
**Bowtie_SAMtools**	43678	651	1521	0.966	0.985	0.976	0.015	3278	361	780	0.808	0.901	0.852	0.099
**BWA_DeepVariant**	43918	411	1345	0.970	0.991	0.980	0.009	3467	172	477	0.879	0.953	0.914	0.047
**BWA_FreeBayes**	43609	720	1591	0.965	0.984	0.974	0.016	3389	250	603	0.849	0.931	0.888	0.069
**BWA_GATK**	43852	477	1591	0.965	0.989	0.977	0.011	3471	168	501	0.874	0.954	0.912	0.046
**BWA_SAMtools**	43893	436	1385	0.969	0.990	0.980	0.010	3219	420	809	0.799	0.885	0.840	0.115
**MOSAIK_DeepVariant**	43502	827	1820	0.960	0.981	0.970	0.019	3381	258	581	0.853	0.929	0.890	0.071
**MOSAIK_FreeBayes**	43590	739	1630	0.964	0.983	0.974	0.017	3331	308	621	0.843	0.915	0.878	0.085

**MOSAIK_GATK**	19436	24893	1023	0.950	0.438	0.600	0.562	3131	508	2601	0.546	0.860	0.668	0.140
**MOSAIK_SAMtools**	-	-	-	-	-	-	-	-	-	-	-	-	-	-
**Novoalign_DeepVariant**	43921	408	1301	0.971	0.991	0.981	0.009	3482	157	471	0.881	0.957	0.917	0.043
**Novoalign_FreeBayes**	43552	777	1721	0.962	0.982	0.972	0.018	3311	328	684	0.829	0.910	0.867	0.090
**Novoalign_GATK**	43841	488	1610	0.965	0.989	0.977	0.011	3475	164	489	0.877	0.955	0.914	0.045
**Novoalign_SAMtools**	43901	428	1399	0.969	0.990	0.980	0.010	3299	340	724	0.820	0.907	0.861	0.093
**SOAP_DeepVariant**	-	-	-	-	-	-	-	-	-	-	-	-	-	-
**SOAP_FreeBayes**	42871	1458	2895	0.937	0.967	0.952	0.033	3201	438	2576	0.554	0.880	0.680	0.120
**SOAP_GATK**	43172	1157	2751	0.940	0.974	0.957	0.026	3099	540	2555	0.548	0.852	0.667	0.148
**SOAP_SAMtools**	-	-	-	-	-	-	-	-	-	-	-	-	-	-
