# Supplementary material for: Performance assessment of variant calling pipelines using human whole exome sequencing and simulated data
Source: BMC Bioinformatics. 2019 Jun 17;20:342. doi: 10.1186/s12859-019-2928-9 (PMC6580603; doi:10.1186/s12859-019-2928-9)
Supplement: Supplementary file 15 — Table S10. Comparison of three benchmarking studies that used GiaB gold standard variant dataset NA12878 (PDF 202 kb) [file 12859_2019_2928_MOESM15_ESM.pdf]

Table S10. Comparison of three benchmarking studies that used GiaB gold standard variant dataset NA12878

	Present study	Hwang et al., 2015	Highnam et al, 2015	Cornish and Guda, 2015
**GiaB Version**	GiaB V3.3.2	GiaB high confident integrated pedigree calls V0.2	GiaB V2.18	GiaB V2.15
**Genome Version**	GRCh37&38	GRCh37	GRCh37	GRCh37
**Sequencing Platform**	HiSeq2500	HiSeq2000	HiSeq2000	HiSeq2000
**Data sets used**	NA12878, NA24385, NA24631 and Simulated Reads(WES)	12 Datasets NA12878(WES, WGS) including IonTorrent	NA12878(WES) and Simulated Reads	NA12878(WES)
**Aligners**	BWA-MEM 0.7.16<br>Bowtie2 2.2.6<br>Novoalign 3.08.02<br>SOAP 2.21<br>MOSAIK 2.2.3	Bowtie2-2.2.25<br>BWA-MEM-0.7.10<br>Novoalign-3.02.12<br>Tmap (for Ion Proton)	Bowtie2-2.0.0-beta5<br>BWA-0.7.5a<br>BWA-MEM-0.7.5a<br>Novoalign-3.00.04	Bowtie2-2.1.0<br>BWA sampe-0.7.5a<br>BWA-MEM-0.7.5a<br>CUSHAW3-3.0.3<br>MOSAIK-2.2.3<br>Novoalign-3.02.07
**Variant callers**	GATK HaplotypeCaller 4<br>FreeBayes 1.1.0<br>SAMtools mpileup 1.7<br>DeepVariant r0.4	FreeBayes-0.9.18<br>GATK-HaplotypeCaller3.3.0<br>SAMtools mpileup-1.1<br>TVC-4.2.3 (for Ion Proton)	GATK-HaplotypeCaller3.1-1<br>GATK-UnifiedGenotyper3.1-1<br>SAMtools mpileup v0.1.18<br>Isaac v01.13.06.20	FreeBayes v9.9.2-19- g011561f<br>GATK-HaplotypeCaller2.7-2<br>GATK-UnifiedGenotyper2.7-2<br>SAMtools mpileup-0.1.19<br>SNPSVM-0.01
**Benchmark Method**	F-Score	Area under Precision-recall curve	Positive predictive value	Positive predictive value, sensitivity separately
**Best Performing Pipeline**	BWA/Novoalign_DeepVariant (SNVs and InDels were analyzed separately)	SNVs: BWA_SAMtools<br>InDels: BWA_GATK HaplotypeCaller<br>(SNVs and InDels were analyzed separately)	Novoalign_GATK HaplotypeCaller (SNV and InDel were combined for this analysis)	Novoalign_GATK HaplotypeCaller (Only SNV was analyzed)
